# Altered Synapse Stability in the Early Stages of Tauopathy

**DOI:** 10.1016/j.celrep.2017.03.013

**Published:** 2017-03-28

**Authors:** Johanna S. Jackson, Jonathan Witton, James D. Johnson, Zeshan Ahmed, Mark Ward, Andrew D. Randall, Michael L. Hutton, John T. Isaac, Michael J. O’Neill, Michael C. Ashby

**Affiliations:** 1 Lilly UK, Erl Wood Manor, Windlesham, Surrey GU20 6PH, UK; 2 Centre for Synaptic Plasticity, School of Physiology, Pharmacology and Neuroscience, University of Bristol, Biomedical Sciences Building, University Walk, Bristol BS8 1TD, UK

**Keywords:** dementia, cortex, 2-photon microscopy, axon, bouton, dendritic spine, neurodegeneration

## Abstract

Synapse loss is a key feature of dementia, but it is unclear whether synaptic dysfunction precedes degenerative phases of the disease. Here, we show that even before any decrease in synapse density, there is abnormal turnover of cortical axonal boutons and dendritic spines in a mouse model of tauopathy-associated dementia. Strikingly, tauopathy drives a mismatch in synapse turnover; postsynaptic spines turn over more rapidly, whereas presynaptic boutons are stabilized. This imbalance between pre- and post-synaptic stability coincides with reduced synaptically driven neuronal activity in pre-degenerative stages of the disease.

## Highlights

• Density of cortical axonal boutons and dendritic spines is reduced early in tauopathy
• Abnormalities in synaptic stability and size exist before decreases in synapse density
• Turnover of dendritic spines is elevated, whereas presynaptic boutons are stabilized
• Neuronal activity is reduced at stages associated with mismatched synaptic turnover

## Graphical Abstract

**Figure:**
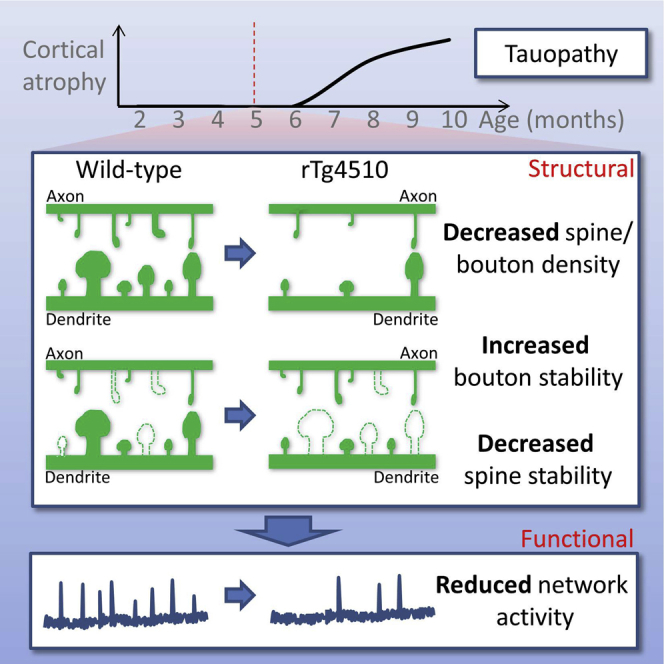

## Teaser

Using in vivo two-photon imaging in the rTg4510 tauopathy mouse model, Jackson et al. find that synapse stability is altered during the pre-degenerative stages of tauopathy. Mismatched abnormalities in pre- and post-synaptic turnover coincide with disrupted neuronal activity.

## Introduction

A major hallmark of neurodegenerative dementia is the loss of neuronal synapses. Indeed, in Alzheimer’s disease, synapse loss is the best-known cellular correlate of cognitive decline (Scheff et al., 2006). The rTg4510 transgenic mouse line, in which the P301L-mutated human tau (*MAPT*) gene that causes Frontotemporal Dementia with Parkinsonism-17 is expressed in excitatory neurons of the forebrain, recapitulates many characteristics of neurodegenerative disease (Ramsden et al., 2005, Santacruz et al., 2005). Specifically, deposition of insoluble neurofibrillary tangles, the hallmark of tauopathy-related dementia, begins in the cortex from ∼5 months of age (Ramsden et al., 2005, Spires et al., 2006). This is followed by age-dependent loss of cortical neurons and progressive forebrain atrophy (Ramsden et al., 2005, Spires et al., 2006). It is known that neocortical dendritic spine and synapse density is decreased late in the disease, at 9–10 months of age (Crimins et al., 2011, Kopeikina et al., 2013, Rocher et al., 2010). However, it was recently shown that neuronal firing patterns are altered as early as 5 months of age, leading to the suggestion that perturbations in coordinated synaptic activity may occur in early-stage pathology (Menkes-Caspi et al., 2015). Most importantly, deficits in cognitive function seem to emerge in advance of major histopathology (Hunsberger et al., 2014), as suggested for dementia (Sperling et al., 2011). Therefore, pursuing the hypothesis that synaptic dysfunction is an early harbinger of dementia, we have determined when and how changes in synapse number are first manifested in rTg4510 mice.

## Results

To measure the dynamics of synapses, we used in vivo two-photon microscopy to repeatedly image dendritic spines and axonal terminaux boutons (TBs) of GFP-expressing pyramidal neurons in the somatosensory cortex of rTg4510 mice and littermate controls. The same regions of interest containing several sections of neurite were imaged weekly (typically three or four imaging sessions per animal). To span several months at weekly intervals, we studied three groups of animals at different ages ranging from early (early, 18–19 weeks/∼4 months old) to intermediate (mid, 21–24 weeks/∼5 months old) to advanced stages, when clear neurodegeneration has taken root in the cortex (late, 26–29 weeks/∼6.5 months old) (Ramsden et al., 2005).

### Pre- and Post-synaptic Components Are Lost as Pathology Progresses in rTg4510 Animals

**Figure 1:**
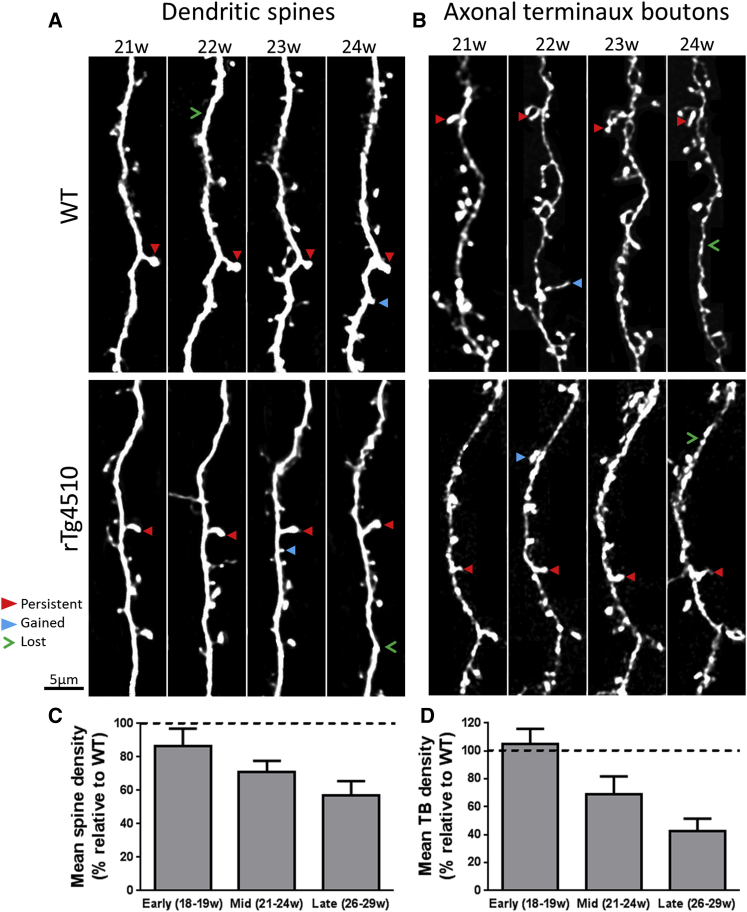
Progressive Simultaneous Loss of Pre- and Post-synaptic Components in rTg4510 Mice (A and B) Dendrites (A) and axons (B) bearing TBs were repeatedly imaged at weekly intervals in WT and rTg4510 animals. Scale bar, 5μm. In some image panels, for clarity of display, fluorescence not associated with main neurite was removed. (C) There is a progressive decrease in spine density with age in rTg4510 dendrites relative to WT (mean ± SEM; early, *F*_(1,54)_ = 0.31, p = 0.580, WT: n = 39 dendrites/5 animals, rTg4510 n = 16/4; mid, *F*_(1,64)_ = 2.07, p = 0.169, WT: n = 8/3, rTg4510 n = 10/3; late, *F*_(1,50)_ = 44.09, p < 0.001, WT n = 15/4, rTg4510 n = 12/3). (D) Quantification of TB loss in the same three batches of animals shows that loss of axonal TBs follows a similar age-dependent decrease relative to WT (mean ± SEM; early, *F*_(1,29)_ = 0.12, p = 0.728, WT:n = 16/3, rTg4510: n = 7/3; mid, *F*_(1,54)_ = 1.92; p = 0.183; WT: n = 13/3, rTg4510: n = 14/3; late, *F*_(1,57)_ = 10.76; p = 0.004; WT: n = 13/4, rTg4510 n = 8/4). Two-way repeated-measures ANOVA. Error bars represent mean ± SEM.

As a potential indicator of synaptic degeneration, we compared the relative density of spines (Figure 1A) and TBs (Figure 1B) in wild-type (WT) and transgenic animals. In the youngest animals, there was no genotype dependence in spine density (Figure 1C; early; p = 0.580). However, relative to WT, there was a progressive decrease in spine density between 4 and 6.5 months in transgenic animals (Figure 1C). As a consequence, at the latest age examined, there was a dramatic relative decrease in spine density in the transgenic cortex (Figure 1C; late; p < 0.001). In the mid group, while the mean relative spine density lay between the early and late values, the effect of genotype was not significant overall (Figure 1C; mid; p = 0.169). Pairwise comparisons at specific imaging weeks within the mid group first reported a significant difference in spine density between genotypes in the last week (24 weeks, p = 0.029), perhaps suggesting that this is a transitional age for the beginnings of spine loss.

The relative density of axonal boutons mirrored the age-dependent decreases in dendritic spine density (Figure 1D). No differences were found relative to WT in the early group (Figure 1D; p = 0.728) or at mid time points (Figure 1D; p = 0.183). However, at the later time points, a significant decrease in bouton density in rTg4510 animals compared to littermate controls (Figure 1D; p = 0.004). These data suggest that the loss of synaptic structures occurs prior to significant cell loss in the cortex of rTg4510 mice (Spires et al., 2006).

### Dendritic Spine Turnover Is Increased but Axonal Bouton Turnover Is Decreased in rTg4510 Animals

**Figure 2:**
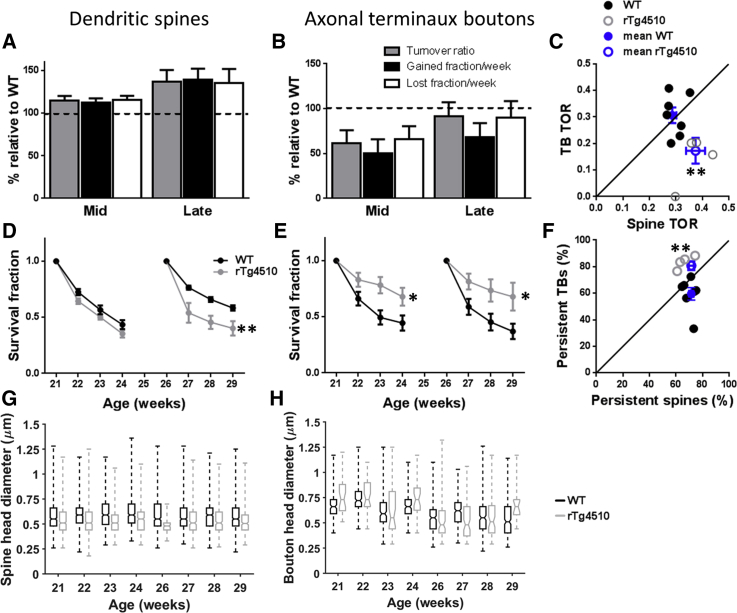
Dissociated Turnover and Altered Stability of Dendritic Spines and Axonal Boutons (A) rTg4510 dendrites had a significantly increased TOR of dendritic spines at mid and late time points driven by a significant increase in lost spines and gained spines at the mid and late time points respectively (mean ± SEM; p = 0.003; mid, WT: n = 16/4, rTg4510 n = 17/4; late, WT: n = 17/4, rTg4510 n = 8/3). (B) rTg4510 axonal TBs showed a significantly reduced turnover at both mid and late time points due to decreases in both gains and losses of TB (p = 0.003; mid, WT: n = 17/3, rTg4510 n = 9/3; late, WT: n = 13/4, rTg4510 n = 9/4). (C) TBs and spines from the same WT individuals showed a balanced turnover (p = 0.786; n = 7 animals). This association was lost in rTg4510s where dendritic spines had a significantly increased TOR compared to axonal boutons (p = 0.007; n = 4 animals). (D) Survival of dendritic spines from the first to last imaging session is decreased in rTg4510s in the late group (*F*_(1,41)_ = 14.87; p < 0.001; WT: n = 17/4, rTg4510: n = 8/3), but not at the mid time points (*F*_(1,46)_ = 5.90; p = 0.053; WT: n = 16/4, rTg4510: n = 17/4). (E) The survival fraction of rTg4510 axonal TBs was increased in the mid group (*F*_(1,46)_ = 5.90; p = 0.023; WT: n = 16/3, rTg4510: n = 8/3) and in the late group (*F*_(1,36)_ = 6.60; p = 0.019; WT: n = 14/4, rTg4510: n = 6/3). (F) The relative proportions of persistent TBs and spines is shifted in rTg4510 animals (p = 0.054; n = 7 animals/group) compared to WT (p = 0.04; n = 4 animals/group). Error bars represent mean ± SEM. (G) Boxplots showing decreased spine head diameter for rTg4510 compared to WT for each imaging week (box, 25^th^/75^th^ percentile; line, median; whiskers, full range; WT n = 4,805 spines, 4 animals, TG n = 3,516 spines, 4 animals). (H) Boxplots showing no change in TB head diameter for rTg4510 compared to WT (box, 25^th^/75^th^ percentile; line, median; whiskers, full range; WT n = 1,608 TBs, 4 animals, TG n = 410 TBs, 4 animals). Two-way repeated-measures ANOVA (A, B, D, and E), Student’s unpaired t test (C and F), and linear mixed model (G and H) were used; ^∗^p < 0.05, ^∗∗^p < 0.01.

Ongoing addition and removal of a small but significant fraction of synapses is thought to underlie the continual tuning of neuronal function to ongoing cognitive demands or in response to injury or disease (Trachtenberg et al., 2002, Holtmaat et al., 2006, Majewska et al., 2006, Cruz-Martín et al., 2010, Grillo et al., 2013, Murmu et al., 2013). Perturbation of synaptic turnover or stability could underlie or indicate altered neuronal function in dementia. To assess how synaptic turnover relates to the progression of tauopathy, we measured weekly gains and losses of spines and TBs in the mid and late age groups (Figure 2).

Turnover of dendritic spines was significantly higher in both age groups in rTg4510 dendrites than in WT dendrites (Figure 2A; p = 0.003). This elevated turnover was driven by a relatively balanced increase in both addition and removal of spines (Figure 2A). Notably, even as overall spine density is decreasing (Figure 1C), rTg4510 animals in the oldest group have elevated addition of new spines relative to WT. In stark contrast, we found that the turnover of rTg4510 axonal TBs was reduced relative to WT due to decreases in both lost and gained boutons at both time points (Figure 2B; p = 0.003).

Comparing spine and TB dynamics within individual animals, we observed that turnover of pre- and post-synaptic elements was relatively balanced in WT animals (Figure 2C; p = 0.786; n = 7 animals/group) but that this balance was lost in rTg4510 animals, as spines were more dynamic whereas axonal boutons became more stable (Figure 2C; p = 0.007; n = 4 animals/group).

### Stability of Both Dendritic Spines and Axonal Boutons Are Affected in Tg4510 Animals

To investigate processes underlying the apparent change in turnover dynamics, we assessed the likelihood of synaptic structures persisting each week from our first imaging session to the last (survival fraction). For spines present at 21 weeks of age, there was no difference between rTg4510 and WT in month-long survival fraction (Figure 2D; p = 0.053; WT: n = 16/4, rTg4510: n = 17/4), but survival was significantly reduced in spines present at 26 weeks of age (Figure 2D; p < 0.001; WT: n = 17/4, rTg4510: n = 8/3). This contrasts with the likelihood of long-term survival of axonal TBs, which was increased in rTg4510s at the mid time points (Figure 2E; p = 0.023; WT: n = 16/3, rTg4510: n = 8/3) and late time points (Figure 2E; p = 0.019; WT: n = 14/4, rTg4510 n = 6/3). Therefore, tauopathy may shift the balance of transient (lasting only 1 week) and persistent (lasting at least 2 weeks) synaptic structures. Indeed, comparing axons and dendrites in the same animal, the proportion of persistent spines and boutons is balanced in WTs (Figure 2F; p = 0.054), but in rTg4510 animals, the axonal boutons are significantly more persistent than the corresponding spine population (Figure 2F; p = 0.004).

### Size of Dendritic Spines, but Not Axonal Boutons, Is Affected in Tg4510 Animals

We reasoned that impaired turnover of synaptic structures may lead to defects in neuronal communication via changes in synapse strength and/or connectivity. In line with this suggestion, we found that the size of dendritic spines, which correlates with synaptic strength (Rochefort and Konnerth, 2012), exhibited a small but consistent reduction in rTg4510 animals compared to controls across all time points (Figure 2G; p < 0.001 for genotype, p = 0.11 for age). Intriguingly, there was no apparent effect of genotype on the size of TBs, again highlighting dissociation between pre- and post-synaptic consequences of tauopathy (Figure 2H; p = 0.1862).

### Spontaneous and Evoked Cortical Neuronal Activity Is Decreased in rTg4510 Animals

**Figure 3:**
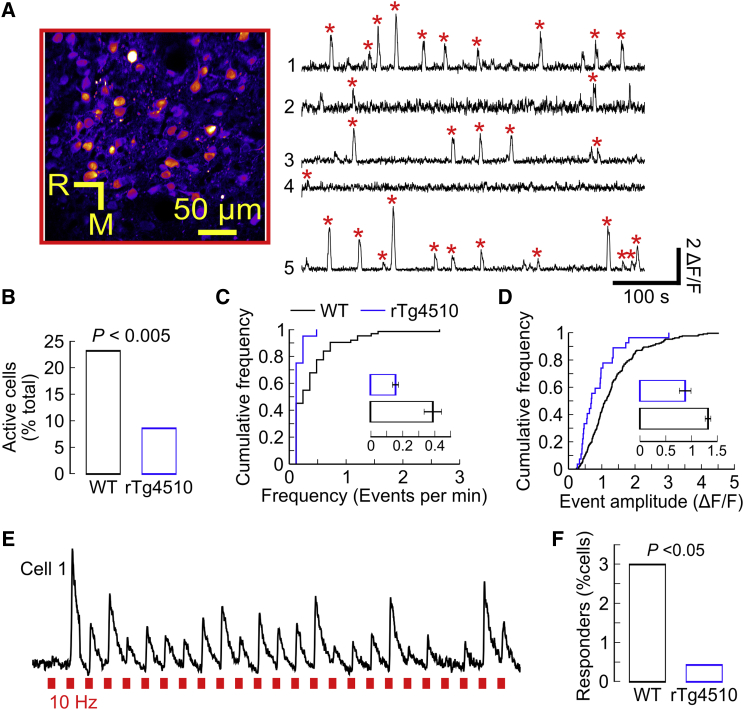
Decreased Cortical Neuronal Activity and Aberrant Stimulus Encoding in rTg4510 Mice (A) Image shows representative field of view showing GCaMP6m fluorescence in layer 2 neurons of barrel cortex (R, rostral; M, medial). Right: example GCaMP6m fluorescence traces (ΔF/F) for five randomly selected neurons. Red stars denote the detection of spontaneous GCaMP6m transients. (B) Percentages of WT (n = 268, 3 mice, 89 ± 14 per mouse [mean ± SEM]) and rTg4510 (n = 233, 3 mice, 78 ± 6 per mouse [mean ± SEM]) neurons with isolated GCaMP6m transients (p = 1e-5, χ^2^_(1)_ = 19.32; WT = 62/268 neurons, rTg4510 = 20/233 neurons). (C) Cumulative frequency and inset bar graph (mean ± SEM) illustrating the frequency of spontaneous GCaMP6m transients in active WT (n = 62) and rTg4510 (n = 20) neurons (p = 0.005, rank-sum test; WT = 0.39 ± 0.05 events per min, rTg4510 = 0.16 ± 0.02 events per min). (D) Cumulative frequency and inset bar graphs (mean ± SEM) illustrating the amplitudes of spontaneous GCaMP6m transients in WT (n = 208 transients, 62 cells) and rTg4510 (n = 27 transients, 20 cells) neurons (p = 0.001, rank-sum test; WT = 1.32 ± 0.06 ΔF/F, rTg4510 = 0.88 ± 0.12 ΔF/F). (E) Representative GCaMP6m transients showing single trial responses to contralateral principal whisker stimulation (10 Hz, 1 s; 25 trials; red dashes). (F) Percentages of WT (n = 268 neurons, three mice) and rTg4510 (n = 233 neurons, three mice) neurons responding to whisker stimulation (p = 0.03, χ^2^_(1)_ = 4.62; WT = 8/268 neurons, rTg4510 = 1/233 neurons).

If reduced spine size reflects a reduction in synaptic strength, we would predict a perturbation of synaptically driven neuronal activity in the early stages of the disease. To test this prediction, we imaged neuronal activity in populations of GCaMP6-expressing layer 2 neurons in the somatosensory cortex of 22-week-old animals. Up to this age point, we had found changes in synaptic turnover but no significant decrease in synapse density. Under a consistent level of light anesthesia (Figure S2), we measured somatic Ca^2+^ transients of individual neurons simultaneously within regions responding to deflection of a defined, single whisker (Figure 3A; see Experimental Procedures). We found that tauopathy was indeed associated with a reduction in the proportion of cells displaying spontaneous (non-stimulated) neuronal activity (Figure 3B; p < 0.005). Furthermore, in active cells, both the frequency (Figure 3C; p = 0.005) and amplitude (Figure 3D; p = 0.001) of spontaneous events were reduced in rTg4510 mice. These data suggest that spontaneous activity is less likely to occur and consists of fewer action potentials when it does. To more directly assess the synaptic drive of neuronal spiking, we deflected the principal whisker while imaging the subsequent neuronal responses. In line with other studies that indicate sparse sensory coding in this neuronal population (Petersen and Crochet, 2013), only a small proportion of cells showed robust responses to stimulation (Figure 3E). However, the proportion of neurons responding was significantly reduced in rTg4510 mice compared to WT, suggesting that early tauopathy does lead to a reduction in coordinated synaptic drive (Figure 3F; p = 0.03).

## Discussion

In dementia and in many animal models of dementia-related neurodegeneration, there are debilitating effects on brain function that appear before, or even in the absence of, major cell loss (Sperling et al., 2011). This implicates aberrant neuronal function in mediating these early symptoms. It has been suggested that loss of synapses that precedes neuronal loss may underlie these early deficits (Scheff et al., 2006, Spires-Jones and Knafo, 2012). In this study, we measured the time course of synapse loss in the cortex during the early stages of tauopathy-driven neurodegeneration. Our findings suggest that synapse number does indeed decrease ahead of when neurons die, with pre-and post-synaptic sites affected at similar stages. The classic histopathological marker of tauopathy are neurofibrillary tangles (NFTs). Aggregated tau can be found in diseased dendrites, axons, and NFTs in the soma (Ludvigson et al., 2011). However, evidence suggests that aggregated tau may not be a crucial factor in synaptic or structural abnormalities, even quite late in tauopathy (Rocher et al., 2010). In accordance, our data suggest that tau-driven synaptic pathologies are evident before much evidence of NFTs is seen in the cortex. As such, it seems likely the mechanisms at play in our findings are pathophysiological rather than aggregate-driven neurotoxicity.

Indeed, we found that that synapse stability and function is altered even before overt synapse loss. Our data show that the early stages of tauopathy are associated with alterations in both pre- and post-synaptic turnover rates. Surprisingly, the effects alter axonal and dendritic dynamics in apparently opposite directions, with enhanced long-term presynaptic stabilization contrasting with elevated postsynaptic plasticity. This mismatch in plasticity on either side of the synapse was entirely unexpected and is, so far, unexplained. It is possible that different mechanisms of tauopathy-driven pathology exist in axonal and dendritic compartments. Axonal tau normally acts to stabilize the microtubule-based cytoskeleton and facilitate fast axonal transport, which may be perturbed in rTg4510 mice. Pharmacological stabilization of microtubules can improve axonal integrity in a mouse model of tauopathy (Brunden et al., 2010), so it is possible that such a perturbation could also affect the dynamics of presynaptic structures. Under normal conditions, tau is not found in large amounts in the dendritic compartment, but it does become mis-localized there under disease conditions (de Calignon et al., 2012, Liu et al., 2012). The pathological signaling mechanisms of dendritic tau could be distinct from those found presynaptically. Indeed, mis-localized tau has been shown to affect neurotransmitter receptor content at synapses (Hoover et al., 2010), which is likely to influence dendritic spine turnover via synaptic plasticity (Sala and Segal, 2014). Interestingly, a recent study found a similar instability of dendritic spines in a mouse model of Huntington’s disease (Murmu et al., 2013). In this model, tau is not explicitly affected, which potentially suggests that spine instability could be a more general consequence of neuronal dysfunction associated with neurodegenerative disease. An alternative possibility is that the opposing effects on synapse stability represent homeostatic responses to pathology initiated on one side of the synapse. For example, elevated postsynaptic turnover could be a response to the lack of plasticity on the presynaptic side. In our experiments, we were not able to simultaneously image both sides of the synaptically coupled cells, but the axons, which are TB rich, likely emanate from layer 6 neurons (De Paola et al., 2006) that do make some synaptic connections in layer 1 on pyramidal neurons (Shepherd et al., 2005).

What are the implications for synaptic anatomy of mismatched turnover rates? Perhaps rapidly turned over spines might not stabilize because they are never acquire a presynaptic partner, or perhaps there is a shift in the number of dendritic partners for multi-release site axonal boutons. Electron microscopy of rTg4510 cortical synapses might provide an answer to this.

We have shown that the tauopathy-associated alterations in synaptic dynamics align with both anatomical and functional indications of reduced synaptic drive. This is manifested as reduced neuronal spiking rates, both spontaneous and those driven by passive whisker deflection (Figure 3). Our findings showing reduced neuronal responsiveness contrast with a previous study showing little effect on visual orientation-tuned responses (Kuchibhotla et al., 2014) or in “resting” calcium levels in the visual cortex of 8- to 10-month-old rTg4510 mice (Kopeikina et al., 2013). Varying regional effects could underlie these differences, but it is perhaps more likely that the very different ages of the animals is an important factor to consider. The present study is focused on an early age that largely precedes neuronal death or degeneration, whereas the visual cortex studies were conducted at a time when tau has become aggregated into NFTs, many neurons have died, and the cortex is significantly atrophied (Ramsden et al., 2005). Cortical function likely undergoes huge plasticity in the face of this age-related degeneration, and direct comparison of pre-pathology cellular phenotypes with those of neurons surviving late into neurodegeneration is very difficult. Another recent study revealed defects in oscillatory network activity and in neuronal membrane potential changes driven by synchronized synaptic activity in young rTg4510 animals (Menkes-Caspi et al., 2015). In line with this, the data presented here suggest that alterations in synapse turnover dynamics, and their potential impact on synaptic function, are likely to influence circuit function and plasticity at the earliest, pre-degenerative stages of tauopathy-related dementia.

## Experimental Procedures

Male rTg4510 mice and WT littermates were injected in somatosensory cortex with adeno-associated virus that drove neuronal expression of either GFP or GCaMP6m. A cranial window was implanted over the injection site to allow in vivo two-photon imaging following expression of the fluorescent protein in superficial cortical layers. For structural imaging, GFP filling the same axonal and dendritic regions was imaged weekly in head-fixed, anesthetized animals to visualize the turnover of presynaptic TBs and postsynaptic dendritic spines. Analysts blind to genotype and age scored the presence and location of individual synaptic structures to allow longitudinal tracking of their turnover. For functional imaging, cell bodies of GCaMP6-expressing layer 2 neurons within the principal whisker column were imaged in lightly anesthetized, head-fixed animals. GCaMP6 fluorescence changes, which correspond to neuronal activity, were measured over time following deflection of the principal whisker to assess synaptically driven activity in these cells. All procedures were conducted by researchers holding a UK personal license and conducted in accordance with the UK Animals (Scientific Procedures) Act 1986 and subject to internal ethical review. Further experimental detail can be found in Supplemental Experimental Procedures.

## Author Contributions

J.S.J., J.W., M.C.A., A.R., M.L.H., M.J.O., and J.I. designed the study; J.S.J. and J.W. carried out the in vivo experiments; J.S.J., J.W., J.D.J., and M.C.A. analyzed in vivo data; and Z.A. and M.W. carried out the histology experiments and data analysis. M.C.A., J.T.I., A.D.R., and M.J.O. jointly supervised the project. All authors contributed to the manuscript preparation.

## Supplemental Information

Document S1. Supplemental Experimental Procedures and Figures S1–S3

Document S2. Article plus Supplemental Information
