# Supplementary material for: Altered Synapse Stability in the Early Stages of Tauopathy
Source: Cell Rep. 2017 Mar 28;18(13):3063–8. doi: 10.1016/j.celrep.2017.03.013 (PMC5382238; doi:10.1016/j.celrep.2017.03.013)
Supplement: Document S1. Supplemental Experimental Procedures and Figures S1–S3 [file mmc1.pdf]

Cell Reports, Volume *18*

Supplemental Information

Altered Synapse Stability in the Early Stages of Tauopathy

Johanna S. Jackson, Jonathan Witton, James D. Johnson, Zeshan Ahmed, Mark Ward, Andrew D. Randall, Michael L. Hutton, John T. Isaac, Michael J. O'Neill, and Michael C. Ashby

SUPPLEMENTARY INFORMATION – "Altered synapse stability in the early stages of tauopathy", Jackson et al.

Supplementary Information Inventory

- Supplementary Experimental Procedures – detailed description of experimental and analytical procedures
- Supplementary Figure 1 (related to Figure 1) – Histopathological data related to potential impact of viral injection and cranial window implantation.
- Supplementary Figure 2 (related to Figure 3) – Example recording of online analysis of breathing rate during functional calcium imaging, and distribution of signal:noise ratio comparing genotypes from experiments in Figure 3.
- Supplementary Figure 3 (related to Figures 1&3) – Generalized Linear Mixed Models to assess the potential impact of "between-animal" differences on structural (related to Figure 1) and functional (related to Figure 3) datasets.

Supplementary Experimental procedures

Surgery, imaging and image analysis were performed blind with respect to genotype and age.

Animals

Adult male mice of the rTg(tau_{301L})4510 (TG; n=14) line and wild-type littermate controls (WT;n=16) were used between 18 and 29 weeks of age (Ramsden et al., 2005). Mice were screened for activator and responder transgenes using a standardized PCR assay (Santacruz et al., 2005). Although this transgene contains a conditional expression cassette, mice were maintained off doxycycline-containing diet throughout, meaning mutant tau was continually expressed (Santacruz et al., 2005). All mice were given ad libitum access to food and water and maintained in a 12 hour light-dark cycle. All procedures were conducted by researchers holding a UK personal licence and conducted in accordance with the UK Animals (Scientific Procedures) Act 1986 and subject to internal ethical review.

Surgery

Cranial windows were surgically implanted over the somatosensory cortex as previously described (Holtmaat et al., 2012). In addition, adeno-associated virus expressing GFP or GCaMP6m was injected into the layer 2/3 of the cerebral cortex to enable the visualisation of cortical neurons. Briefly, mice were anaesthetized with isofluorane and administered dexamethasone (30 mg/kg) to limit brain swelling and the analgesic, buprenorphine (5 mg/kg) pre-operatively. The skull was exposed and a 5 mm diameter craniotomy drilled over the somatosensory cortex. For neurite imaging experiments AAV serotype 2 expressing GFP (10^{10} GU; Vector Biolabs), or, for neuronal activity imaging experiments, AAV (1/2 hybrid serotype) containing GCaMP6m (Chen et al., 2013) under control of the human synapsin promoter (UPenn Vector Core) was injected into layer 2/3 of the cerebral cortex (3 injection sites, 0.3µl per site, 300µm below the dura). A glass coverslip was placed over the craniotomy and sealed with glue and dental cement. A screw was placed in the skull on the contralateral side for added stability. The whole skull was subsequently covered in dental cement and a stainless steel head-post (<500 mg) was affixed to the skull contralateral to the cranial window using bone cement for positioning on the two-photon microscope. Mice were allowed to recover for at least 3 weeks before imaging began. Pilot studies showed that the injected virus diffused in the cortex and transduced cells in all cortical layers in both groups of animals.

Immunohistochemistry

Brains were coronally dissected into three segments using an adult mouse brain matrix (slot #5 and #11 AP; RBM-2000C: ASI Instruments, USA). These segments were processed using the Tissue TEK® VIP processor (GMI Inc, USA) and embedded in paraffin wax. The middle segment was used to cut 8 µm serial sections using rotary microtomes (HM 200 and HM 355; Thermo Scientific, Germany) which were mounted on glass slides. Coronal sections representing approximately Bregma -1.50 (AP) were selected for immunohistochemistry using primary antibodies specific for phospho-tau (PG-5, tau phosphorylated at s409; 1:8000, from

Peter Davies) and microglia (Iba-1: 1:6000, Wako Chemicals GmbH, Germany). Following de-paraffinisation and rehydration of the tissue, antigen retrieval was performed using the Lab Vision PT module system (Thermo Scientific, UK), where sections were heated to 100°C for 20 min in citrate buffer (TA-250-PM1X; Thermo Scientific, UK). After cooling in dH_2O, the slides were transferred to the Lab Vision Autostainer 360 (Thermo Scientific, UK) where the following incubations were performed: 10 min in H2O2 (0.03%); 30 min in normal goat serum (1:20; Vector Laboratories, USA); 60 min in primary antibodies; 30 min in biotinylated goat anti-mouse or anti-rabbit IgG (1:200, PA-920 or BA-1000; Vector Laboratories, USA); 30 min avidin-biotin complex solution (PK-7100; Vector Laboratories, USA); 5 min in 3,3′-diaminobenzidine (SK-4105; Vector Laboratories, USA). Apart from the last two steps, PBS with 0.05% Tween-20 (PBS-T) was used for diluting reagents and washes between steps. Sections were then counterstained with haematoxylin before dehydration and cover-slipping.

Neuropathological analysis

Stained sections were digitised using the Scanscope XT slide scanner (Aperio, USA) at 20× magnification. Imagescope software (version 11.1.2.760; Aperio, USA) was used to view the digitised tissue sections and delineate boundaries of the hippocampus and overlying cortex (including barrel field of the somatosensory cortex) for both the right (cranial window) and left (contralateral) side of brain. Immunoreactivity for PG-5 positive tau pathology and Iba-1 positive microglia within the regions of interest was quantified using the positive pixel algorithm (Imagescope, version 11.1.2.760; Aperio, CA, USA) and expressed as a percentage of the total area. Levels of Iba-1 and PG-5 in cortical regions were normalized to their respective levels in the hippocampus. Injection and cranial window implantation did trigger an increase in microglial activation, measured by Iba-1 immunostaining, in the ipsilateral cortex. However, this both WT and rTg4510 mice were equally prone to this elevation (**Figure S1**).

There was no difference in tau burden in cortex ipsilateral to the cranial window compared to the contralateral hemisphere (**Figure S1**).

Two-photon imaging of neurite structure

Three groups of animals were imaged; an early group imaged between 18 and 19 weeks of age, a middle group imaged between 21 and 25 weeks, and a later group imaged between 25 and 29 weeks. All time points are prior to the onset of widespread cell death in the cortex.

A two-photon microscope (Prairie Technologies) equipped with a tuneable Ti:Sapphire pulsed laser (~100 fs pulsewidth, MaiTai HP, SpectraPhysics) and PrairieView acquisition software was used for all imaging experiments. Mice were anaesthetized with 3-5% isoflurane and secured to the microscope with the metal bar attached to the skull to a custom-built fixed support. Body temperature of 35-37°C was maintained by a heating blanket and rectal probe. A 10x objective (NA=0.3, Olympus) was used to identify characteristic blood vessels to reliably locate regions-of-interest (ROIs) at each imaging time point. A 40x water immersion objective (LUMPlanFI/IR, NA=0.8, Olympus) was used to acquire several ROI stacks (75 μm x 75 μm, 512 x 512 pixels, step size = 0.5 μm) per animal. Excitation power at 910 nm was typically 35mW at the sample. GFP emission was collected through a 525/25nm filter. At the first imaging session, 5-10 ROIs of axons and/or dendrites were acquired ensuring that the ROIs were at least 100 μm apart. Animals underwent subsequent imaging sessions, each lasting approximately one hour, up to a maximum of 20 sessions. The signal-to-noise ratio was measured in WT and rTg4510 images and no significant difference was found.

Widefield epifluorescence imaging of neuronal activity

22 week-old mice were head-fixed under sedation (2 mg/kg cholorprothixene) and light, constant anaesthesia (0.4-0.8 % isoflurane). Body temperature of 35-37°C was maintained by a heating blanket and rectal probe. Barrel locations were mapped in S1 via widefield imaging

of neuronal GCaMP6m fluorescence (Minderer et al., 2012). Individual whiskers were loaded into a 19 gauge cannula to approximately 5 mm from the snout, and deflected caudo-rostrally (5° deflections, 10 Hz, 2 s) using a piezo bending actuator (Piezo Systems, Woburn, MA). Neuronally-expressed GCaMP6m was excited by illuminating the cortical surface with 480 nm light and epifluorescence images were captured through a 500-550nm bandpass emission filter at 10 Hz using a 4x objective (Plan N, Olympus, NA 0.1) and 12-bit CCD camera (QICAM Fast 1394, QImaging). Stimulation-evoked responses were calculated per pixel per trial (10 trials, 20 s inter-trial interval) as the fractional change in mean GCaMP6m fluorescence 0-2 s post whisker-stimulation onset relative a 0-2 s pre-stimulation baseline. Response fields were determined as the largest contiguous area in which the in which the response amplitude exceeded 50% of the maximum response. Regions within the response field were subsequently targeted for higher magnification two-photon imaging.

Two-photon imaging of neuronal activity

Images were acquired at 40x objective magnification (LUMPlanFI/IR, 0.8 NA, Olympus) using a two-photon microscope (Scientifica), equipped with Ti:sapphire pulsed laser (~100 fs pulsewidth, Mai Tai-HP, Newport Spectra-Physics) and galvanometric scan mirrors. GCaMP6m was excited at 910 nm and fluorescence collected using a 525/25 nm emission filter. Excitation power was <35mW at the sample Images were acquired using bidirectional frame scanning at 8 Hz, 256 x 128 pixel resolution, using ScanImage 3.8 software (Pologruto et al., 2003). Superficial cortical blood vessels were used as fiducial markers to guide imaging to regions as close as possible to the principal whisker response field that contained a high density of GCaMP6m expressing layer 2/3 neurons (100-300 µm below pial surface) and did not overlap with major blood vessels. A high-resolution (0.15 µm per pixel) reference image was acquired of each imaging field to assist post-hoc definition of neuronal regions of interest (ROIs). Spontaneous GCaMP6m signals were recorded continuously for 520 s. For whisker

stimulation experiments, images were acquired continuously while the principal whisker was deflected caudo-rostrally (5° deflections, 10 Hz, 1 s) at a 20 s inter-stimulus interval across 25 trials using a piezo actuator. To ensure that a consistent depth of anaesthesia was maintained throughout and between experiments, breathing rate was continuously monitored using a piezoelectric wafer beneath the thorax, and anaesthesia adjusted to maintain a rate >100 breaths per min (**Figure S2B-D**) (Golshani and Portera-Cailliau, 2008). In separate experiments, we determined that small variations within the range of breathing rates used here (100-140 breaths per min) did not correlate with the rate or amplitude of spontaneous neuronal activity. Deeper anaesthesia, and associated slower breathing rate, dramatically reduced apparent excitability (data not shown).

<u>Spine and bouton structure analysis</u>

In vivo two-photon images were converted into stacks with ImageJ and the StackReg plugin run to align the stacks in case of any movement. The stacks were deconvolved with Huygens Deconvolution software using a quick maximum likelihood estimation with an experimentally defined point-spread-function. Deconvolved stacks were then imported into Neurolucida and the density of dendritic spines or axonal boutons determined. To confirm that a fluorescent protrusion was attached to the dendritic/axonal shaft, the brightness and contrast of the image was adjusted to see a visible spine/bouton neck. Analysts distinguished between dendritic spines and axonal TBs by their attachment to a thicker straighter dendritic shaft compared to the thinner, more tortuous, axonal process, as described in the existing literature (De Paola et al., 2006; Majewska et al., 2006). No changes were observed in the proportion of dendritic or axonal filopodia at any time point (data not shown). For analysis of turnover, the image stacks were imported into ImageJ and spines or boutons marked using the Cell Counter plugin for each imaging session. These parameters were then further analysed in Microsoft Excel and statistics carried out in SigmaPlot. Turnover ratio (TOR) was determined by TOR (t_1,t_2) =

$(N_{gained} + N_{lost})/N(t_1) + N(t_2)$ where $N(t_1)$ and $N(t_2)$ are the total number of synaptic structures at the first and second time point respectively. Survival fraction was calculated as $SF(t) = N(t)/N_0$ where N_0 is the number of structures at t=0 and N(t) if the number of structures of present at the first imaging session surviving after time t. Persistent and transient spines/boutons were defined as ones there for at least two imaging sessions (>8days) or only one imaging session (<8 days) respectively. For animals where axons and dendrites were imaged, the TOR, gains and losses and persistent fraction was compared, using a Student's *t* test, between axons and dendrites in both WT and rTg4510 groups. Two-way repeated measures ANOVA tests were used with the Holm-Sidak method of pairwise comparison in all analyses to determine significance when $p < 0.05$ and where n was the number of axons or dendrites. Only those axons/dendrites which were present for two imaging sessions at the early time point and >3 imaging sessions in the mid and late time points were used. The size of TB or spine heads was measured as the diameter of a manually-drawn circle that bounded the widest part of the structure (in deconvolved image stacks). Individual measurements were grouped based on age and genotype and statistically tested using a Generalized Linear Model (SPSS, IBM).

Neuronal activity analysis

Analyses were performed using Matlab (Mathworks) and ImageJ software (National Institutes of Health). Mechanical drift was corrected by registration to the average of the first 5 seconds (40 frames) using the TurboReg plugin (Thevenaz et al., 1998) in ImageJ. Summed and standard deviation projections of time-lapse images were used to manually draw ROIs around visually identified neuronal cell bodies.

For analysis of spontaneous GCaMP6m signals, neuronal fluorescence time series, *F(t),* were constructed by averaging the ROI pixels in each frame. The background time series was

determined from a reference blood vessel and was subtracted from *F(t)*. Relative time series were generated following the procedure of Jia et al. (Jia et al., 2011). Briefly, a time-varying baseline, $F_0(t)$, was determined by first smoothing *F(t)* with a ±1 s moving average filter to generate *F'(t),* and then for each time point, *t*, F_0 was calculated as the minimum of *F'(t)* in the preceding 5 second epoch, such that:

$$F_0(t) = \{\min(\mathrm{F'(x)}) \mid (t-5) < x < t\}$$

where $F'(x) = \frac{1}{2}\int_{x-1}^{x+1} F(\tau)\mathrm{d}(\tau)$

The relative fluorescence time series was then calculated as:

$$R(t) = \frac{F(t) - F_0(t)}{F_0(t)}$$

Finally, the relative time series was smoothed with an exponentially weighted moving average to generate *ΔF/F*:

$$\Delta F/F(t) = \frac{\int_0^t R(t-\tau)\cdot w(\tau)\mathrm{d}\tau}{\int_0^t w(\tau)\mathrm{d}\tau}$$

where $w(\tau) = exp\left\{-\frac{|\tau|}{0.5}\right\}$

This routine is robust against slow drifts in baseline fluorescence (Jia et al., 2011).

Spontaneous fluorescence transients were isolated using a high-throughput template-based event detection algorithm, following the methodology of Clements and Bekkers (Clements and Bekkers, 1997). A template, *T*, was constructed by averaging and smoothing (0.5 s moving average) the waveforms of 310 manually approved events (aligned to event onset) from WT neurons (n=3 mice, 5 sessions). The minima and maxima of *T* were normalised to 0 and 1

respectively. For each ΔF/F time series, *T* was advanced point-by-point along the time series and the fit, *Q*, calculated as:

$$Q = T * S + P$$

where *T* is the template, *S* is the template scaling factor, and *P* is the offset:

$$S = \frac{\sum(T * D) - \sum T * \sum D/N}{\sum T^2 - \sum T * \sum T/N}$$

$$P = \frac{\sum D - S * \sum T}{N}$$

where N is the number of samples in the template, Σ is the sum from 1 to N, and *D* is the period of the ΔF/F time series to fit, such that:

$$D = \{\Delta\, \mathrm{F/F(x)} \mid t \le x \le (t + N - 1)\}$$

The detection criterion, *C*, at each time point, *t*, was then calculated from *S* and the standard error of the fit, *E*:

$$E = \sqrt{\frac{\sum(D - Q)^2}{N - 1}}$$

such that

$$C(t) = \frac{S}{E}$$

This algorithm directly relates the detection criterion to the signal-to-noise ratio of the fit, thus accounting for variable levels of noise within and between neuronal time series (Clements and Bekkers, 1997). Events were detected at times, *t*, when *C(t)* exceeded a threshold of 2. Event rise times were calculated using the 20-80 % portion of the initial rising phase of the event.

For analysis of whisker stimulation-evoked responses, neuronal fluorescence time series were constructed and background corrected. The time series were binned into 10 s whisker

stimulation trial epochs (WS) and interleaved 10 s non-whisker stimulation trial epochs (NS), and relative fluorescence time series computed for individual WS and NS with baseline fluorescence, F_0, dynamically calculated as the mean of the 2 s pre stimulus period, such that:

$$\Delta F/F_0(t) = \frac{F(t)-F_0}{F_0}$$

Neuronal WS responses were defined as the integral of the ΔF/F time series 0-2 s post whisker stimulation onset. Neurons were classified as responsive to the whisker stimulus if, (1) the distribution of WS responses was significantly greater than the distribution of NS responses (determined using a 2-sample KS-test), and (2) the peak amplitude of the trial averaged response (0-2 post stimulation) exceeded 4 times the standard deviation of the trial averaged baseline (0-2 pre stimulation). This stringent detection criterion generated zero false positives, based on post-hoc visual inspection of the individual WS and NS traces.

Comparison of the signal-to-noise ratio of image regions from individual cells (calculated following the protocol of Kerlin et al. (2010)) showed that there was no difference in the population distribution between WT and rTg4510 animals (**Figure S2A**). This suggests that the imaging preparation and GCaMP6m expression are comparable across genotype.

Statistical testing was performed using SPSS (IBM) and Matlab. Datasets were tested for normality and parametric or nonparametric tests used as appropriate with an α level of 0.05. Data are presented as mean ± SEM unless otherwise stated. n numbers for statistical testing are noted as “n=…” throughout.

Statistical analysis of effects of individual animals

In all statistical analyses (except Figures 2C&F, where spine and TB results are compared within animals), the cell (or cellular events in Figure 3D) was chosen as our primary dependent variable (or “n” number). This level of analysis was used (in particular, avoiding averaging

within each animal) because we were particularly interested in the variability between individual cells or neurites. This is because neurodegeneration is a slowly progressing process that affects different neurons at different times. It is changes in the characteristics of this population distribution that are likely to be a primary indicator of potential disease effects. However, cells within an individual animal are not independent and may have a tendency to co-vary, which could generate bias within our datasets. To minimize potential difficulties due to unevenly weighted contributions of individual animals, balanced numbers of cells/neurites from each animal were included in each sample. Further, to formally test the effect of the animal on our datasets, Generalized Linear Mixed Models (GLMM, SPSS) were generated that include "Genotype" as a Fixed factor and "Animal" as a Random factor, allowing by-neuron random intercept, and by-animal random slope and by-genotype random slope. When repeated measures were made (eg, in longitudinal spine density measures), "Age" was also included as a Fixed factor, using a first-order autoregressive (AR1) covariance structure because we anticipate higher covariance between ages that are closer in time. Axon/spine density (**Figure 1**) and cellular activity (**Figure 3**) values were modelled in the GLMM with a gamma distribution and log link function, since population distributions tended to be skewed to higher values.

Results of GLMMs of our structural (spine and TB density) and functional (spontaneous activity) datasets are presented in **Figure S3**. The effects of genotype in the GLMM are established by the "genotype coefficient" (**Figure S3A**), which indicates the average effect of being Tg4510-positive on spine or TB density. For example, Tg4510 dendrites in the late age group will, on average, have 0.594 spines/μm^{-1} less than WT counterparts (**Figure S3A**). The variance in the GLMM that is due to animal-related effects is measured by the "variance due to animal" metric. Significance values for these predicted effects are shown in the final two columns of **Figure S3A** (green highlights $p<0.05$). In line with analyses presented in Figure 1,

significant decreases due to the transgene are found in spine density in the mid and late age groups (**Figure S3B**) and in TB density in the late group (**Figure S3C**). In none of the datasets was there a significant effect of within-animal co-variance (**Figure S3A-C**). Similar GLMMs were used to analyse the spontaneous neuronal calcium elevations recorded in vivo (shown in **Figure 3A-D**). In these analyses, being Tg4510-positive is associated with a significant reduction in the frequency, ampltiude and rise time of spontaneous events (**Figure S3D-G**). Again, in these data, the effect of individual animal identity had no significant influence on the GLMM predictions (**Figure S3D-G**). This quantitatively defines the contribution of animal-to-animal variability as having a limited impact on our findings. We were not able to obtain well-fitted GLMMs for the binomial data for response to whisker stimulation (**Figure 3E-F**), possibly because the fraction of responding cells is relatively small due to the sparse coding of such sensory stimuli in this neuronal population.

Supplementary References

Clements, J.D., and Bekkers, J.M. (1997). Detection of spontaneous synaptic events with an optimally scaled template. Biophys. J. *73*, 220–229.

De Paola, V., Holtmaat, A., Knott, G., Song, S., Wilbrecht, L., Caroni, P., and Svoboda, K. (2006). Cell type-specific structural plasticity of axonal branches and boutons in the adult neocortex. Neuron *49*, 861–875.

Golshani, P., and Portera-Cailliau, C. (2008). In Vivo 2-Photon Calcium Imaging in Layer 2/3 of Mice. J. Vis. Exp. JoVE.

Holtmaat, A., de Paola, V., Wilbrecht, L., Trachtenberg, J.T., Svoboda, K., and Portera-Cailliau, C. (2012). Imaging neocortical neurons through a chronic cranial window. Cold Spring Harb. Protoc. *2012*, 694–701.

Jia, H., Rochefort, N.L., Chen, X., and Konnerth, A. (2011). In vivo two-photon imaging of sensory-evoked dendritic calcium signals in cortical neurons. Nat. Protoc. *6*, 28–35.

Kerlin, A.M., Andermann, M.L., Berezovskii, V.K., and Reid, R.C. (2010). Broadly Tuned Response Properties of Diverse Inhibitory Neuron Subtypes in Mouse Visual Cortex. Neuron *67*, 858–871.

Majewska, A.K., Newton, J.R., and Sur, M. (2006). Remodeling of Synaptic Structure in Sensory Cortical Areas In Vivo. J. Neurosci. *26*, 3021–3029.

Minderer, M., Liu, W., Sumanovski, L.T., Kügler, S., Helmchen, F., and Margolis, D.J. (2012). Chronic imaging of cortical sensory map dynamics using a genetically encoded calcium indicator. J. Physiol. *590*, 99–107.

Pologruto, T.A., Sabatini, B.L., and Svoboda, K. (2003). ScanImage: flexible software for operating laser scanning microscopes. Biomed. Eng. Online *2*, 13.

Ramsden, M., Kotilinek, L., Forster, C., Paulson, J., McGowan, E., SantaCruz, K., Guimaraes, A., Yue, M., Lewis, J., Carlson, G., et al. (2005). Age-dependent neurofibrillary tangle formation, neuron loss, and memory impairment in a mouse model of human tauopathy (P301L). J. Neurosci. *25*, 10637–10647.

Santacruz, K., Lewis, J., Spires, T., Paulson, J., Kotilinek, L., Ingelsson, M., Guimaraes, A., DeTure, M., Ramsden, M., McGowan, E., et al. (2005). Tau suppression in a neurodegenerative mouse model improves memory function. Science *309*, 476–481.

Thevenaz, P., Ruttimann, U.E., and Unser, M. (1998). A pyramid approach to subpixel registration based on intensity. IEEE Trans. Image Process. *7*, 27–41.

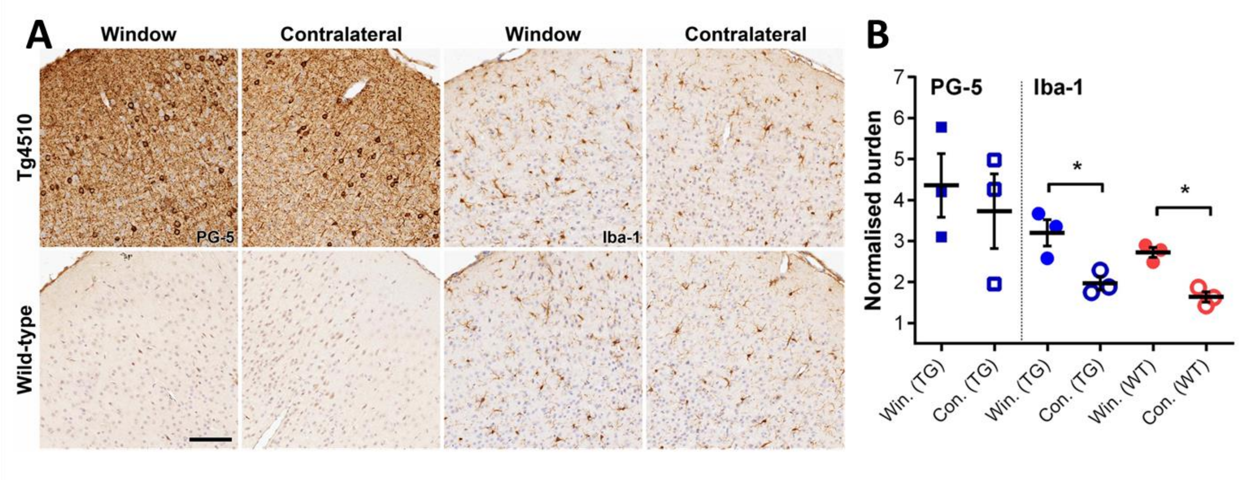

Figure S1 (related to Figure 1): Virus injection and/or cranial window implantation does not cause abnormal pathology in rTg4510 animals at six weeks post-surgery.

(A) Representative images of phosphorylated tau expression (PG5) in the somatosensory cortex beneath the window and in the contralateral hemisphere in WT and rTg4510 animals (left four panels). Representative images of microglial (Iba-1) response to the virus injection and cranial window implantation compared to the contralateral hemisphere in both groups of animals (right four panels). (B) No significant difference was seen in phosphorylated tau levels between the ipsilateral and contralateral hemispheres. However, the somatosensory cortex beneath the window did have a significant increase in microgliosis compared to the contralateral hemisphere in both groups of animals ($F_{(3,8)} = 12.38$; $p=0.002$; WT: $n=3$, rTg4510: $n=3$), B(PG-5): One-way ANOVA, B(Iba-1): Student's unpaired t test. *p<0.05. Error bars = mean ± SEM. Scale bar = 100μm.

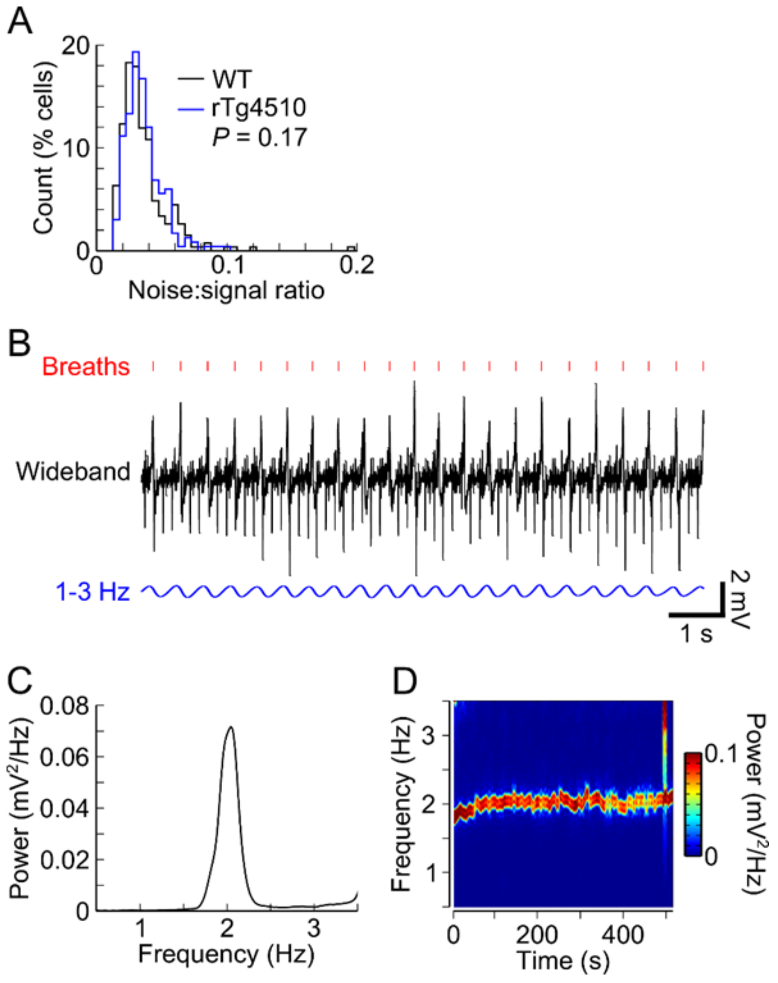

Figure S2 (related to Figure 3): Imaging conditions were consistent throughout functional calcium imaging experiments and between genotypes.

(A) Histograms illustrating consistent fluorescence noise-to-signal ratios between WT (n=268, 3 mice) and rTg4510 (n=233, 3 mice) GCaMP6m-expressing neurons imaged during two-photon imaging experiments (p=0.17; rank-sum test). Ratios were determined by estimating the contribution of photon shot noise to the mean neuronal fluorescence signal, as described by Kerlin et al. (2010). **(B)** Representative 10 s wideband (black) and 1-3 Hz bandpass filtered (blue) traces of breathing movements recorded in a WT mouse during a two-photon imaging experiment using a piezoelectric transducer beneath the thorax. Large amplitude transients (red ticks) correspond to individual breaths. Smaller amplitude, higher frequency transients correspond to heart beats. **(C)** Power spectral density of the wideband trace in (a) between 0.5-

3.5 Hz, illustrating a 2 Hz breathing rate. **(D)** Spectrogram of the full breathing movement trace in (a) across a 520 s time-lapse imaging experiment. A stable ~2 Hz breathing rate was maintained throughout the experiment.

A

dataset		Tg4510 coefficient	variance due to animal	genotype p value	animal p value
spines	Early	-0.167	0.056	0.18	0.16
spines	Mid	-0.374	0.056	0.001	0.054
spines	Late	-0.594	0.117	0.000045	0.082
TBs	Early	0.002	0.057	0.99	0.099
TBs	Mid	-0.243	0.237	0.417	0.142
TBs	Late	-0.819	0.264	0.02	0.137

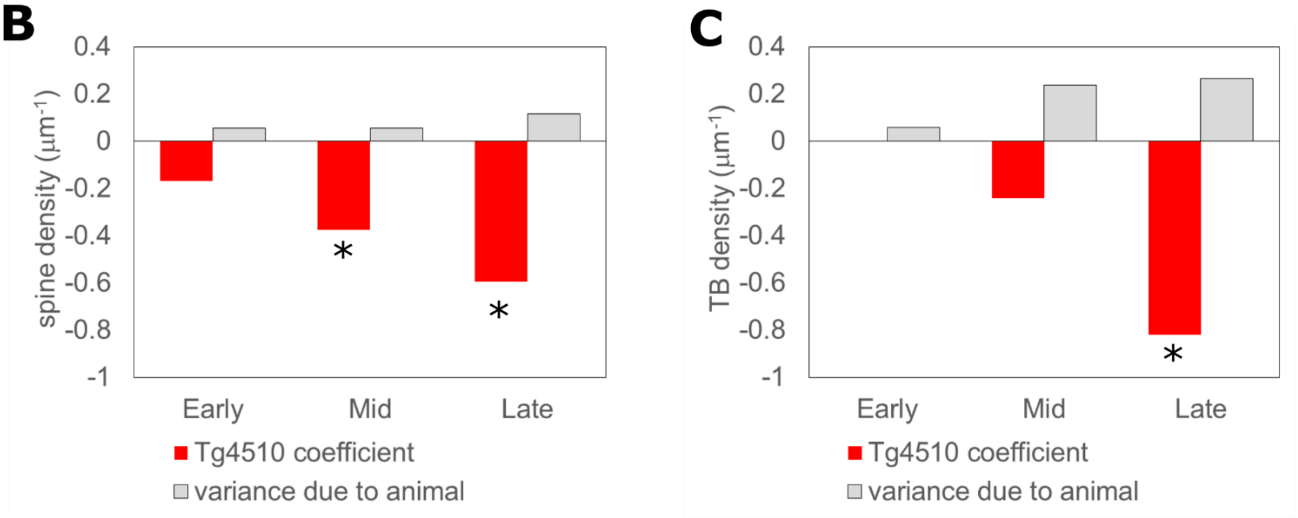

D

analysis	Tg4510 coefficient	variance due to animal	genotype p value	animal p value
event frequency (Hz)	-1.957	0.372	0.0003	0.18
event amplitude ($\Delta F/F_0$)	-0.182	0.072	0.016	0.403
event rise time (ms)	-0.413	0.129	0.018	0.888

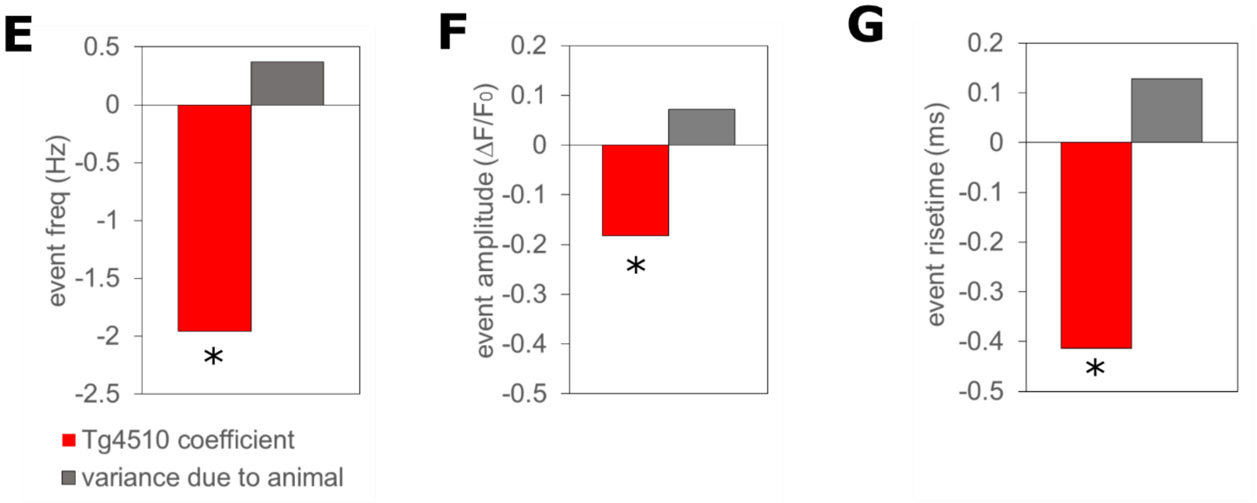

Figure S3 (related to Figures 1&3): Results of GLMM showing that animal-to-animal variance has small and non-significant effects on the primary measures

(A) Table showing the results of GLMM of synapse density for dendritic spines and axonal TBs^{--} in the early, mid and late age groups. Coefficients of genotype (effect of being Tg4510-positive relative to WT) and average variance associated with animal are shown, alongside p values. Dataset as in Figure 1. (B) Histograms showing the effect of being Tg4510-positive on spine density, alongside variance associated with the animal. (C) Histogram showing the effect of being Tg4510-positive on TB density, alongside variance associated with the animal. (D) Table showing the results of GLMMs of properties (frequency, amplitude, rise time) of spontaneous calcium elevations (dataset as in Figure 3A-D). Coefficients of genotype and average variance associated with animal are shown, alongside p values. (E-G) Histograms showing the showing the effect of being Tg4510-positive on the frequency (E), amplitude (F) and rise time (G) of spontaneous calcium events, alongside variance associated with the animal in each case. (* indicates $p<0.05$, highlighted in green in tables). (E)
