# Supplementary material for: Altered Synapse Stability in the Early Stages of Tauopathy
Source: Cell Rep. 2017 Mar 28;18(13):3063–8. doi: 10.1016/j.celrep.2017.03.013 (PMC5382238; doi:10.1016/j.celrep.2017.03.013)
Supplement: Document S2. Article plus Supplemental Information [file mmc2.pdf]

Cell Reports

Report

Altered Synapse Stability in the Early Stages of Tauopathy

Graphical Abstract

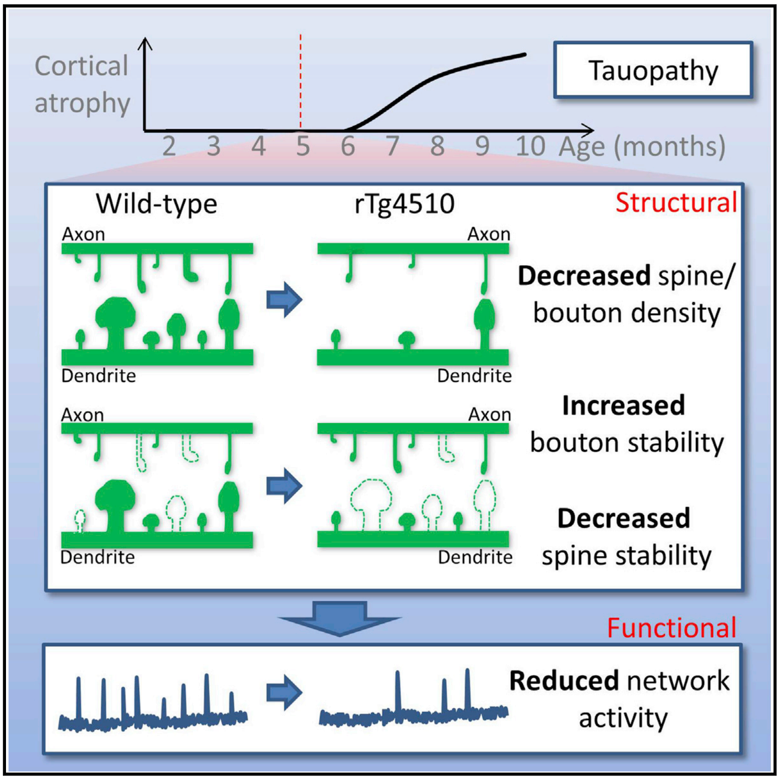

Highlights

- Density of cortical axonal boutons and dendritic spines is reduced early in tauopathy
- Abnormalities in synaptic stability and size exist before decreases in synapse density
- Turnover of dendritic spines is elevated, whereas presynaptic boutons are stabilized
- Neuronal activity is reduced at stages associated with mismatched synaptic turnover

Authors

Johanna S. Jackson, Jonathan Witton, James D. Johnson, ..., John T. Isaac, Michael J. O'Neill, Michael C. Ashby

Correspondence

m.c.ashby@bristol.ac.uk

In Brief

Using in vivo two-photon imaging in the rTg4510 tauopathy mouse model, Jackson et al. find that synapse stability is altered during the pre-degenerative stages of tauopathy. Mismatched abnormalities in pre- and post-synaptic turnover coincide with disrupted neuronal activity.

Jackson et al., 2017, Cell Reports *18*, 3063–3068
March 28, 2017 © 2017 The Author(s).
http://dx.doi.org/10.1016/j.celrep.2017.03.013

Cell Reports
Report

OPEN ACCESS
CellPress

Altered Synapse Stability in the Early Stages of Tauopathy

Johanna S. Jackson,[1,3] Jonathan Witton,[2,3] James D. Johnson,[1,2] Zeshan Ahmed,[1] Mark Ward,[1] Andrew D. Randall,[2,4] Michael L. Hutton,[1] John T. Isaac,[1,5] Michael J. O'Neill,[1] and Michael C. Ashby[2,6,*]
[1]Lilly UK, Erl Wood Manor, Windlesham, Surrey GU20 6PH, UK
[2]Centre for Synaptic Plasticity, School of Physiology, Pharmacology and Neuroscience, University of Bristol, Biomedical Sciences Building, University Walk, Bristol BS8 1TD, UK
[3]Co-first author
[4]Present address: Institute of Biomedical and Clinical Sciences, University of Exeter Medical School, Hatherly Laboratories, University of Exeter, Exeter EX4 4PS, UK
[5]Present address: Neuroscience at the J&J London Innovation Centre, One Chapel Place, London, W1G 0BG, UK
[6]Lead Contact
*Correspondence: m.c.ashby@bristol.ac.uk
http://dx.doi.org/10.1016/j.celrep.2017.03.013

SUMMARY

Synapse loss is a key feature of dementia, but it is unclear whether synaptic dysfunction precedes degenerative phases of the disease. Here, we show that even before any decrease in synapse density, there is abnormal turnover of cortical axonal boutons and dendritic spines in a mouse model of tauopathy-associated dementia. Strikingly, tauopathy drives a mismatch in synapse turnover; postsynaptic spines turn over more rapidly, whereas presynaptic boutons are stabilized. This imbalance between pre- and post-synaptic stability coincides with reduced synaptically driven neuronal activity in pre-degenerative stages of the disease.

INTRODUCTION

A major hallmark of neurodegenerative dementia is the loss of neuronal synapses. Indeed, in Alzheimer's disease, synapse loss is the best-known cellular correlate of cognitive decline (Scheff et al., 2006). The rTg4510 transgenic mouse line, in which the P301L-mutated human tau (*MAPT*) gene that causes Frontotemporal Dementia with Parkinsonism-17 is expressed in excitatory neurons of the forebrain, recapitulates many characteristics of neurodegenerative disease (Ramsden et al., 2005; Santacruz et al., 2005). Specifically, deposition of insoluble neurofibrillary tangles, the hallmark of tauopathy-related dementia, begins in the cortex from ~5 months of age (Ramsden et al., 2005; Spires et al., 2006). This is followed by age-dependent loss of cortical neurons and progressive forebrain atrophy (Ramsden et al., 2005; Spires et al., 2006). It is known that neocortical dendritic spine and synapse density is decreased late in the disease, at 9–10 months of age (Crimins et al., 2011; Kopeikina et al., 2013; Rocher et al., 2010). However, it was recently shown that neuronal firing patterns are altered as early as 5 months of age, leading to the suggestion that perturbations in coordinated synaptic activity may occur in early-stage pathology (Menkes-Caspi et al., 2015). Most importantly, deficits in cognitive function seem to emerge in advance of major histopathology (Hunsberger et al., 2014), as suggested for dementia (Sperling et al., 2011). Therefore, pursuing the hypothesis that synaptic dysfunction is an early harbinger of dementia, we have determined when and how changes in synapse number are first manifested in rTg4510 mice.

RESULTS

To measure the dynamics of synapses, we used in vivo two-photon microscopy to repeatedly image dendritic spines and axonal terminaux boutons (TBs) of GFP-expressing pyramidal neurons in the somatosensory cortex of rTg4510 mice and littermate controls. The same regions of interest containing several sections of neurite were imaged weekly (typically three or four imaging sessions per animal). To span several months at weekly intervals, we studied three groups of animals at different ages ranging from early (early, 18–19 weeks/~4 months old) to intermediate (mid, 21–24 weeks/~5 months old) to advanced stages, when clear neurodegeneration has taken root in the cortex (late, 26–29 weeks/~6.5 months old) (Ramsden et al., 2005).

Pre- and Post-synaptic Components Are Lost as Pathology Progresses in rTg4510 Animals

As a potential indicator of synaptic degeneration, we compared the relative density of spines (Figure 1A) and TBs (Figure 1B) in wild-type (WT) and transgenic animals. In the youngest animals, there was no genotype dependence in spine density (Figure 1C; early; $p = 0.580$). However, relative to WT, there was a progressive decrease in spine density between 4 and 6.5 months in transgenic animals (Figure 1C). As a consequence, at the latest age examined, there was a dramatic relative decrease in spine density in the transgenic cortex (Figure 1C; late; $p < 0.001$). In the mid group, while the mean relative spine density lay between the early and late values, the effect of genotype was not significant overall (Figure 1C; mid; $p = 0.169$). Pairwise comparisons

This is an open access article under the CC BY license (http://creativecommons.org/licenses/by/4.0/).

OPEN
ACCESS
CellPress

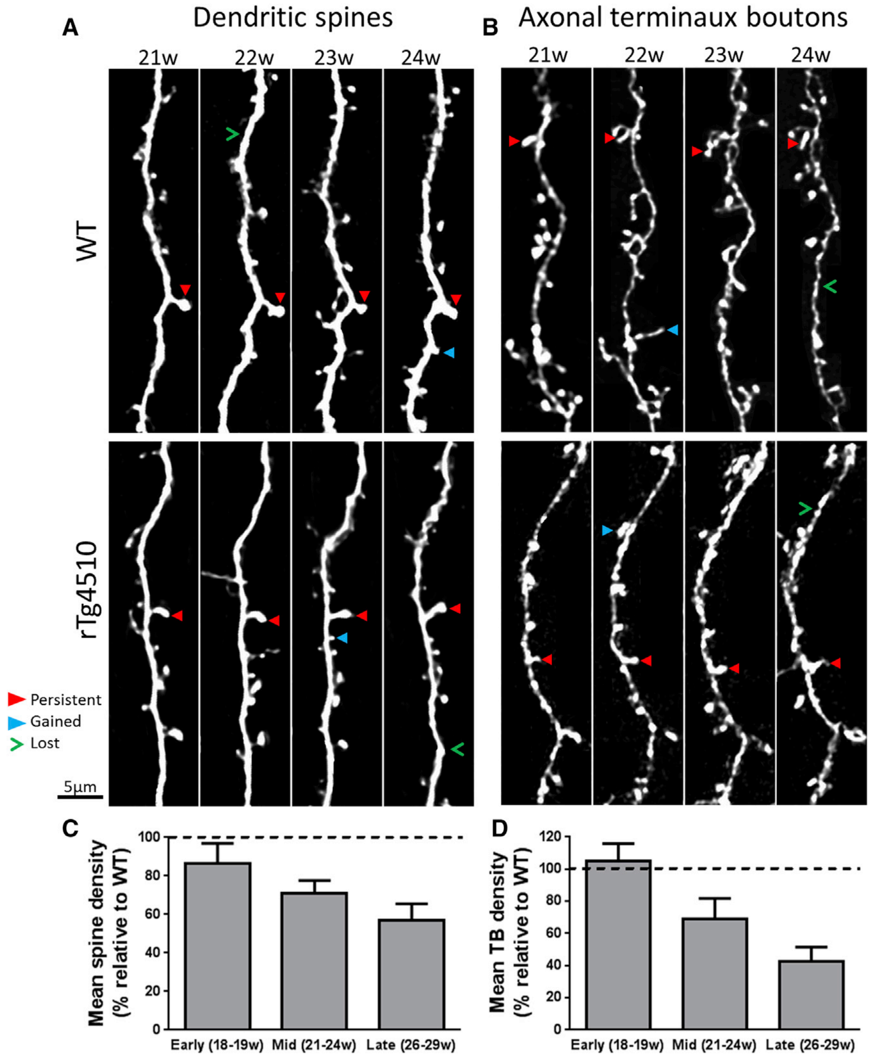

Figure 1. Progressive Simultaneous Loss of Pre- and Post-synaptic Components in rTg4510 Mice

(A and B) Dendrites (A) and axons (B) bearing TBs were repeatedly imaged at weekly intervals in WT and rTg4510 animals. Scale bar, 5μm. In some image panels, for clarity of display, fluorescence not associated with main neurite was removed.

(C) There is a progressive decrease in spine density with age in rTg4510 dendrites relative to WT (mean ± SEM; early, $F_{(1,54)} = 0.31$, p = 0.580, WT: n = 39 dendrites/5 animals, rTg4510 n = 16/4; mid, $F_{(1,64)} = 2.07$, p = 0.169, WT: n = 8/3, rTg4510 n = 10/3; late, $F_{(1,50)} = 44.09$, p < 0.001, WT n = 15/4, rTg4510 n = 12/3).

(D) Quantification of TB loss in the same three batches of animals shows that loss of axonal TBs follows a similar age-dependent decrease relative to WT (mean ± SEM; early, $F_{(1,29)} = 0.12$, p = 0.728, WT:n = 16/3, rTg4510: n = 7/3; mid, $F_{(1,54)} = 1.92$; p = 0.183; WT: n = 13/3, rTg4510: n = 14/3; late, $F_{(1,57)} = 10.76$; p = 0.004; WT: n = 13/4, rTg4510 n = 8/4). Two-way repeated-measures ANOVA. Error bars represent mean ± SEM.

at specific imaging weeks within the mid group first reported a significant difference in spine density between genotypes in the last week (24 weeks, p = 0.029), perhaps suggesting that this is a transitional age for the beginnings of spine loss.

The relative density of axonal boutons mirrored the age-dependent decreases in dendritic spine density (Figure 1D). No differences were found relative to WT in the early group (Figure 1D; p = 0.728) or at mid time points (Figure 1D; p = 0.183). However, at the later time points, a significant decrease in bouton density in rTg4510 animals compared to littermate controls (Figure 1D; p = 0.004). These data suggest that the loss of synaptic structures occurs prior to significant cell loss in the cortex of rTg4510 mice (Spires et al., 2006).

Dendritic Spine Turnover Is Increased but Axonal Bouton Turnover Is Decreased in rTg4510 Animals

Ongoing addition and removal of a small but significant fraction of synapses is thought to underlie the continual tuning of neuronal function to ongoing cognitive demands or in response to injury or disease (Trachtenberg et al., 2002; Holtmaat et al., 2006; Majewska et al., 2006; Cruz-Martín et al., 2010; Grillo et al., 2013; Murmu et al., 2013). Perturbation of synaptic turnover or stability could underlie or indicate altered neuronal function in dementia. To assess how synaptic turnover relates to the progression of tauopathy, we measured weekly gains and losses of spines and TBs in the mid and late age groups (Figure 2).

Turnover of dendritic spines was significantly higher in both age groups in rTg4510 dendrites than in WT dendrites (Figure 2A; p = 0.003). This elevated turnover was driven by a relatively balanced increase in both addition and removal of spines (Figure 2A). Notably, even as overall spine density is decreasing (Figure 1C), rTg4510 animals in the oldest group have elevated addition of new spines relative to WT. In stark contrast, we found that the turnover of rTg4510 axonal TBs was reduced relative to WT due to decreases in both lost and gained boutons at both time points (Figure 2B; p = 0.003).

Comparing spine and TB dynamics within individual animals, we observed that turnover of pre- and post-synaptic elements was relatively balanced in WT animals (Figure 2C; p = 0.786; n = 7 animals/group) but that this balance was lost in rTg4510 animals, as spines were more dynamic whereas axonal boutons became more stable (Figure 2C; p = 0.007; n = 4 animals/group).

Stability of Both Dendritic Spines and Axonal Boutons Are Affected in Tg4510 Animals

To investigate processes underlying the apparent change in turnover dynamics, we assessed the likelihood of synaptic structures persisting each week from our first imaging session to the

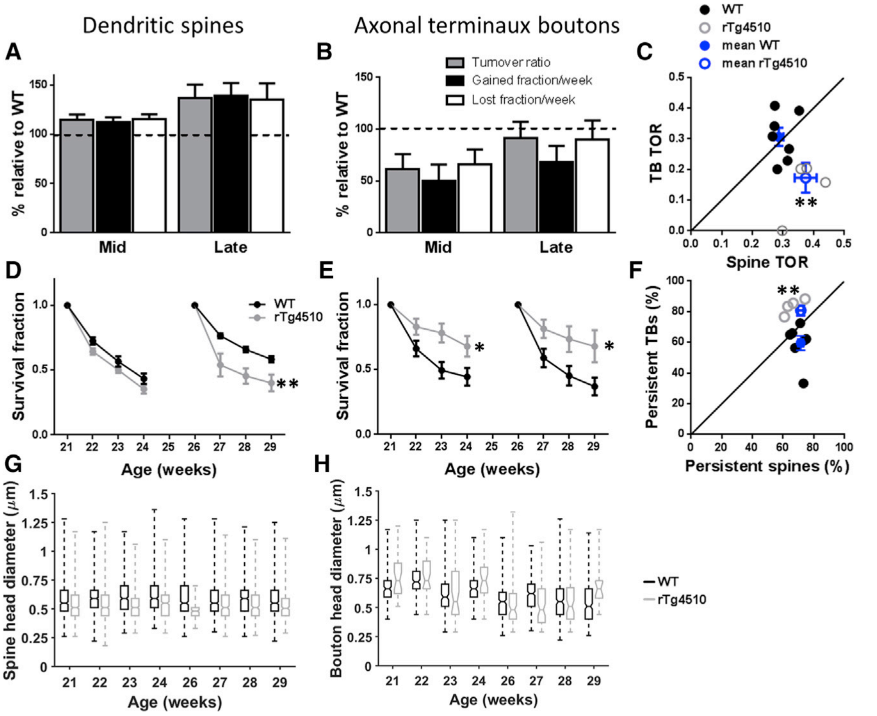

Figure 2. Dissociated Turnover and Altered Stability of Dendritic Spines and Axonal Boutons

(A) rTg4510 dendrites had a significantly increased TOR of dendritic spines at mid and late time points driven by a significant increase in lost spines and gained spines at the mid and late time points respectively (mean ± SEM; p = 0.003; mid, WT: n = 16/4, rTg4510 n = 17/4; late, WT: n = 17/4, rTg4510 n = 8/3).

(B) rTg4510 axonal TBs showed a significantly reduced turnover at both mid and late time points due to decreases in both gains and losses of TB (p = 0.003; mid, WT: n = 17/3, rTg4510 n = 9/3; late, WT: n = 13/4, rTg4510 n = 9/4).

(C) TBs and spines from the same WT individuals showed a balanced turnover (p = 0.786; n = 7 animals). This association was lost in rTg4510s where dendritic spines had a significantly increased TOR compared to axonal boutons (p = 0.007; n = 4 animals).

(D) Survival of dendritic spines from the first to last imaging session is decreased in rTg4510s in the late group ($F_{(1,41)}$ = 14.87; p < 0.001; WT: n = 17/4, rTg4510: n = 8/3), but not at the mid time points ($F_{(1,46)}$ = 5.90; p = 0.053; WT: n = 16/4, rTg4510: n = 17/4).

(E) The survival fraction of rTg4510 axonal TBs was increased in the mid group ($F_{(1,46)}$ = 5.90; p = 0.023; WT: n = 16/3, rTg4510: n = 8/3) and in the late group ($F_{(1,36)}$ = 6.60; p = 0.019; WT: n = 14/4, rTg4510: n = 6/3).

(F) The relative proportions of persistent TBs and spines is shifted in rTg4510 animals (p = 0.054; n = 7 animals/group) compared to WT (p = 0.04; n = 4 animals/group). Error bars represent mean ± SEM.

(G) Boxplots showing decreased spine head diameter for rTg4510 compared to WT for each imaging week (box, $25^{th}/75^{th}$ percentile; line, median; whiskers, full range; WT n = 4,805 spines, 4 animals, TG n = 3,516 spines, 4 animals).

(H) Boxplots showing no change in TB head diameter for rTg4510 compared to WT (box, $25^{th}/75^{th}$ percentile; line, median; whiskers, full range; WT n = 1,608 TBs, 4 animals, TG n = 410 TBs, 4 animals).

Two-way repeated-measures ANOVA (A, B, D, and E), Student's unpaired t test (C and F), and linear mixed model (G and H) were used; *p < 0.05, **p < 0.01.

last (survival fraction). For spines present at 21 weeks of age, there was no difference between rTg4510 and WT in month-long survival fraction (Figure 2D; p = 0.053; WT: n = 16/4, rTg4510: n = 17/4), but survival was significantly reduced in spines present at 26 weeks of age (Figure 2D; p < 0.001; WT: n = 17/4, rTg4510: n = 8/3). This contrasts with the likelihood of long-term survival of axonal TBs, which was increased in rTg4510s at the mid time points (Figure 2E; p = 0.023; WT: n = 16/3, rTg4510: n = 8/3) and late time points (Figure 2E; p = 0.019; WT: n = 14/4, rTg4510 n = 6/3). Therefore, tauopathy may shift the balance of transient (lasting only 1 week) and persistent (lasting at least 2 weeks) synaptic structures. Indeed, comparing axons and dendrites in the same animal, the proportion of persistent spines and boutons is balanced in WTs (Figure 2F; p = 0.054), but in rTg4510 animals, the axonal boutons are significantly more persistent than the corresponding spine population (Figure 2F; p = 0.004).

Size of Dendritic Spines, but Not Axonal Boutons, Is Affected in Tg4510 Animals

We reasoned that impaired turnover of synaptic structures may lead to defects in neuronal communication via changes in synapse strength and/or connectivity. In line with this suggestion, we found that the size of dendritic spines, which correlates with synaptic strength (Rochefort and Konnerth, 2012), exhibited a small but consistent reduction in rTg4510 animals compared to controls across all time points (Figure 2G; p < 0.001 for genotype, p = 0.11 for age). Intriguingly, there was no apparent effect of genotype on the size of TBs, again highlighting dissociation between pre- and post-synaptic consequences of tauopathy (Figure 2H; p = 0.1862).

Spontaneous and Evoked Cortical Neuronal Activity Is Decreased in rTg4510 Animals

If reduced spine size reflects a reduction in synaptic strength, we would predict a perturbation of synaptically driven neuronal activity in the early stages of the disease. To test this prediction, we imaged neuronal activity in populations of GCaMP6-expressing layer 2 neurons in the somatosensory cortex of 22-week-old animals. Up to this age point, we had found changes in synaptic turnover but no significant decrease in synapse density. Under a consistent level of light anesthesia (Figure S2), we measured somatic Ca^{2+} transients of individual neurons simultaneously within regions responding to deflection of a defined, single whisker (Figure 3A; see Experimental Procedures). We found that tauopathy was indeed associated with a reduction in the proportion of cells displaying spontaneous (non-stimulated) neuronal activity (Figure 3B; p < 0.005). Furthermore, in active cells, both the frequency (Figure 3C; p = 0.005) and amplitude (Figure 3D; p = 0.001) of spontaneous events were reduced in

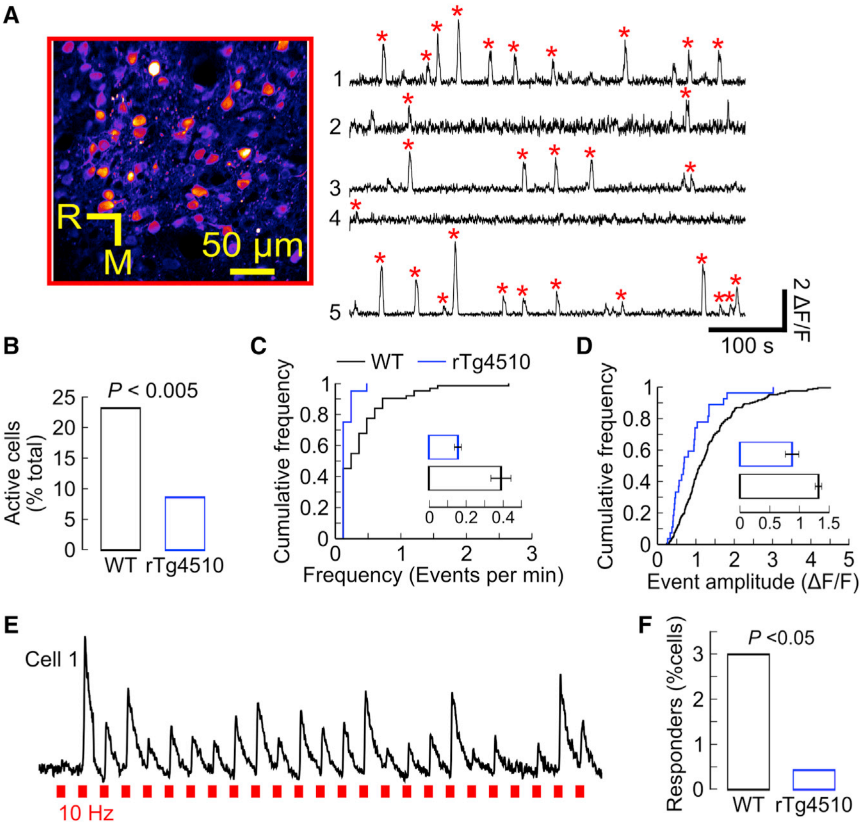

Figure 3. Decreased Cortical Neuronal Activity and Aberrant Stimulus Encoding in rTg4510 Mice

(A) Image shows representative field of view showing GCaMP6m fluorescence in layer 2 neurons of barrel cortex (R, rostral; M, medial). Right: example GCaMP6m fluorescence traces (ΔF/F) for five randomly selected neurons. Red stars denote the detection of spontaneous GCaMP6m transients.

(B) Percentages of WT (n = 268, 3 mice, 89 ± 14 per mouse [mean ± SEM]) and rTg4510 (n = 233, 3 mice, 78 ± 6 per mouse [mean ± SEM]) neurons with isolated GCaMP6m transients (p = 1e-5, $\chi^2_{(1)}$ = 19.32; WT = 62/268 neurons, rTg4510 = 20/233 neurons).

(C) Cumulative frequency and inset bar graph (mean ± SEM) illustrating the frequency of spontaneous GCaMP6m transients in active WT (n = 62) and rTg4510 (n = 20) neurons (p = 0.005, rank-sum test; WT = 0.39 ± 0.05 events per min, rTg4510 = 0.16 ± 0.02 events per min).

(D) Cumulative frequency and inset bar graphs (mean ± SEM) illustrating the amplitudes of spontaneous GCaMP6m transients in WT (n = 208 transients, 62 cells) and rTg4510 (n = 27 transients, 20 cells) neurons (p = 0.001, rank-sum test; WT = 1.32 ± 0.06 ΔF/F, rTg4510 = 0.88 ± 0.12 ΔF/F).

(E) Representative GCaMP6m transients showing single trial responses to contralateral principal whisker stimulation (10 Hz, 1 s; 25 trials; red dashes).

(F) Percentages of WT (n = 268 neurons, three mice) and rTg4510 (n = 233 neurons, three mice) neurons responding to whisker stimulation (p = 0.03, $\chi^2_{(1)}$ = 4.62; WT = 8/268 neurons, rTg4510 = 1/233 neurons).

rTg4510 mice. These data suggest that spontaneous activity is less likely to occur and consists of fewer action potentials when it does. To more directly assess the synaptic drive of neuronal spiking, we deflected the principal whisker while imaging the subsequent neuronal responses. In line with other studies that indicate sparse sensory coding in this neuronal population (Petersen and Crochet, 2013), only a small proportion of cells showed robust responses to stimulation (Figure 3E). However, the proportion of neurons responding was significantly reduced in rTg4510 mice compared to WT, suggesting that early tauopathy does lead to a reduction in coordinated synaptic drive (Figure 3F; p = 0.03).

DISCUSSION

In dementia and in many animal models of dementia-related neurodegeneration, there are debilitating effects on brain function that appear before, or even in the absence of, major cell loss (Sperling et al., 2011). This implicates aberrant neuronal function in mediating these early symptoms. It has been suggested that loss of synapses that precedes neuronal loss may underlie these early deficits (Scheff et al., 2006; Spires-Jones and Knafo, 2012). In this study, we measured the time course of synapse loss in the cortex during the early stages of tauopathy-driven neurodegeneration. Our findings suggest that synapse number does indeed decrease ahead of when neurons die, with pre-and post-synaptic sites affected at similar stages. The classic histopathological marker of tauopathy are neurofibrillary tangles (NFTs). Aggregated tau can be found in diseased dendrites, axons, and NFTs in the soma (Ludvigson et al., 2011). However, evidence suggests that aggregated tau may not be a crucial factor in synaptic or structural abnormalities, even quite late in tauopathy (Rocher et al., 2010). In accordance, our data suggest that tau-driven synaptic pathologies are evident before much evidence of NFTs is seen in the cortex. As such, it seems likely the mechanisms at play in our findings are pathophysiological rather than aggregate-driven neurotoxicity.

Indeed, we found that that synapse stability and function is altered even before overt synapse loss. Our data show that the early stages of tauopathy are associated with alterations in both pre- and post-synaptic turnover rates. Surprisingly, the effects alter axonal and dendritic dynamics in apparently opposite directions, with enhanced long-term presynaptic stabilization contrasting with elevated postsynaptic plasticity. This mismatch in plasticity on either side of the synapse was entirely unexpected and is, so far, unexplained. It is possible that different mechanisms of tauopathy-driven pathology exist in axonal and dendritic compartments. Axonal tau normally acts to stabilize the microtubule-based cytoskeleton and facilitate fast axonal transport, which may be perturbed in rTg4510

mice. Pharmacological stabilization of microtubules can improve axonal integrity in a mouse model of tauopathy (Brunden et al., 2010), so it is possible that such a perturbation could also affect the dynamics of presynaptic structures. Under normal conditions, tau is not found in large amounts in the dendritic compartment, but it does become mis-localized there under disease conditions (de Calignon et al., 2012; Liu et al., 2012). The pathological signaling mechanisms of dendritic tau could be distinct from those found presynaptically. Indeed, mis-localized tau has been shown to affect neurotransmitter receptor content at synapses (Hoover et al., 2010), which is likely to influence dendritic spine turnover via synaptic plasticity (Sala and Segal, 2014). Interestingly, a recent study found a similar instability of dendritic spines in a mouse model of Huntington's disease (Murmu et al., 2013). In this model, tau is not explicitly affected, which potentially suggests that spine instability could be a more general consequence of neuronal dysfunction associated with neurodegenerative disease. An alternative possibility is that the opposing effects on synapse stability represent homeostatic responses to pathology initiated on one side of the synapse. For example, elevated postsynaptic turnover could be a response to the lack of plasticity on the presynaptic side. In our experiments, we were not able to simultaneously image both sides of the synaptically coupled cells, but the axons, which are TB rich, likely emanate from layer 6 neurons (De Paola et al., 2006) that do make some synaptic connections in layer 1 on pyramidal neurons (Shepherd et al., 2005).

What are the implications for synaptic anatomy of mismatched turnover rates? Perhaps rapidly turned over spines might not stabilize because they are never acquire a presynaptic partner, or perhaps there is a shift in the number of dendritic partners for multi-release site axonal boutons. Electron microscopy of rTg4510 cortical synapses might provide an answer to this.

We have shown that the tauopathy-associated alterations in synaptic dynamics align with both anatomical and functional indications of reduced synaptic drive. This is manifested as reduced neuronal spiking rates, both spontaneous and those driven by passive whisker deflection (Figure 3). Our findings showing reduced neuronal responsiveness contrast with a previous study showing little effect on visual orientation-tuned responses (Kuchibhotla et al., 2014) or in "resting" calcium levels in the visual cortex of 8- to 10-month-old rTg4510 mice (Kopeikina et al., 2013). Varying regional effects could underlie these differences, but it is perhaps more likely that the very different ages of the animals is an important factor to consider. The present study is focused on an early age that largely precedes neuronal death or degeneration, whereas the visual cortex studies were conducted at a time when tau has become aggregated into NFTs, many neurons have died, and the cortex is significantly atrophied (Ramsden et al., 2005). Cortical function likely undergoes huge plasticity in the face of this age-related degeneration, and direct comparison of pre-pathology cellular phenotypes with those of neurons surviving late into neurodegeneration is very difficult. Another recent study revealed defects in oscillatory network activity and in neuronal membrane potential changes driven by synchronized synaptic activity in young rTg4510 animals (Menkes-Caspi et al., 2015). In line with this, the data presented here suggest that alterations in synapse turnover dynamics, and their potential impact on synaptic function, are likely to influence circuit function and plasticity at the earliest, pre-degenerative stages of tauopathy-related dementia.

EXPERIMENTAL PROCEDURES

Male rTg4510 mice and WT littermates were injected in somatosensory cortex with adeno-associated virus that drove neuronal expression of either GFP or GCaMP6m. A cranial window was implanted over the injection site to allow in vivo two-photon imaging following expression of the fluorescent protein in superficial cortical layers. For structural imaging, GFP filling the same axonal and dendritic regions was imaged weekly in head-fixed, anesthetized animals to visualize the turnover of presynaptic TBs and postsynaptic dendritic spines. Analysts blind to genotype and age scored the presence and location of individual synaptic structures to allow longitudinal tracking of their turnover. For functional imaging, cell bodies of GCaMP6-expressing layer 2 neurons within the principal whisker column were imaged in lightly anesthetized, head-fixed animals. GCaMP6 fluorescence changes, which correspond to neuronal activity, were measured over time following deflection of the principal whisker to assess synaptically driven activity in these cells. All procedures were conducted by researchers holding a UK personal license and conducted in accordance with the UK Animals (Scientific Procedures) Act 1986 and subject to internal ethical review. Further experimental detail can be found in Supplemental Experimental Procedures.

SUPPLEMENTAL INFORMATION

Supplemental Information includes Supplemental Experimental Procedures and three figures and can be found with this article online at http://dx.doi.org/10.1016/j.celrep.2017.03.013.

AUTHOR CONTRIBUTIONS

J.S.J., J.W., M.C.A., A.R., M.L.H., M.J.O., and J.I. designed the study; J.S.J. and J.W. carried out the in vivo experiments; J.S.J., J.W., J.D.J., and M.C.A. analyzed in vivo data; and Z.A. and M.W. carried out the histology experiments and data analysis. M.C.A., J.T.I., A.D.R., and M.J.O. jointly supervised the project. All authors contributed to the manuscript preparation.

ACKNOWLEDGMENTS

J.D.J. is funded by a BBSRC CASE PhD studentship. Experiments completed in M.C.A.'s laboratory were funded by an MRC centenary award and ARUK Research Fellowship (ARUK-RF2015-6) to J.W., Alzheimer's Research UK (ARUK-ESG2014-3), the Medical Research Council (MR/J013188/1), and EUFP7 Marie Curie Actions (PCIG10-GA-2011-303680). The authors would like to thank Anna Gawedzka, who helped with the image analysis; Jane Cooper, who assisted with the structural imaging; and the University of Bristol Statistics Helpdesk for advice on mixed-model statistical approaches.

Received: July 14, 2016
Revised: October 22, 2016
Accepted: March 1, 2017
Published: March 28, 2017

REFERENCES

Brunden, K.R., Zhang, B., Carroll, J., Yao, Y., Potuzak, J.S., Hogan, A.-M.L., Iba, M., James, M.J., Xie, S.X., Ballatore, C., et al. (2010). Epothilone D improves microtubule density, axonal integrity, and cognition in a transgenic mouse model of tauopathy. J. Neurosci. *30*, 13861–13866.

Crimins, J.L., Rocher, A.B., Peters, A., Shultz, P., Lewis, J., and Luebke, J.I. (2011). Homeostatic responses by surviving cortical pyramidal cells in neurodegenerative tauopathy. Acta Neuropathol. *122*, 551–564.

Cruz-Martín, A., Crespo, M., and Portera-Cailliau, C. (2010). Delayed stabilization of dendritic spines in fragile X mice. J. Neurosci. *30*, 7793–7803.

de Calignon, A., Polydoro, M., Suárez-Calvet, M., William, C., Adamowicz, D.H., Kopeikina, K.J., Pitstick, R., Sahara, N., Ashe, K.H., Carlson, G.A., et al. (2012). Propagation of tau pathology in a model of early Alzheimer's disease. Neuron *73*, 685–697.

De Paola, V., Holtmaat, A., Knott, G., Song, S., Wilbrecht, L., Caroni, P., and Svoboda, K. (2006). Cell type-specific structural plasticity of axonal branches and boutons in the adult neocortex. Neuron *49*, 861–875.

Grillo, F.W., Song, S., Teles-Grilo Ruivo, L.M., Huang, L., Gao, G., Knott, G.W., Maco, B., Ferretti, V., Thompson, D., Little, G.E., and De Paola, V. (2013). Increased axonal bouton dynamics in the aging mouse cortex. Proc. Natl. Acad. Sci. USA *110*, E1514–E1523.

Holtmaat, A., Wilbrecht, L., Knott, G.W., Welker, E., and Svoboda, K. (2006). Experience-dependent and cell-type-specific spine growth in the neocortex. Nature *441*, 979–983.

Hoover, B.R., Reed, M.N., Su, J., Penrod, R.D., Kotilinek, L.A., Grant, M.K., Pitstick, R., Carlson, G.A., Lanier, L.M., Yuan, L.-L., et al. (2010). Tau mislocalization to dendritic spines mediates synaptic dysfunction independently of neurodegeneration. Neuron *68*, 1067–1081.

Hunsberger, H.C., Rudy, C.C., Weitzner, D.S., Zhang, C., Tosto, D.E., Knowlan, K., Xu, Y., and Reed, M.N. (2014). Effect size of memory deficits in mice with adult-onset P301L tau expression. Behav. Brain Res. *272*, 181–195.

Kopeikina, K.J., Polydoro, M., Tai, H.-C., Yaeger, E., Carlson, G.A., Pitstick, R., Hyman, B.T., and Spires-Jones, T.L. (2013). Synaptic alterations in the rTg4510 mouse model of tauopathy. J. Comp. Neurol. *521*, 1334–1353.

Kuchibhotla, K.V., Wegmann, S., Kopeikina, K.J., Hawkes, J., Rudinskiy, N., Andermann, M.L., Spires-Jones, T.L., Bacskai, B.J., and Hyman, B.T. (2014). Neurofibrillary tangle-bearing neurons are functionally integrated in cortical circuits in vivo. Proc. Natl. Acad. Sci. USA *111*, 510–514.

Liu, L., Drouet, V., Wu, J.W., Witter, M.P., Small, S.A., Clelland, C., and Duff, K. (2012). Trans-synaptic spread of tau pathology in vivo. PLoS ONE *7*, e31302.

Ludvigson, A.E., Luebke, J.I., Lewis, J., and Peters, A. (2011). Structural abnormalities in the cortex of the rTg4510 mouse model of tauopathy: a light and electron microscopy study. Brain Struct. Funct. *216*, 31–42.

Majewska, A.K., Newton, J.R., and Sur, M. (2006). Remodeling of synaptic structure in sensory cortical areas in vivo. J. Neurosci. *26*, 3021–3029.

Menkes-Caspi, N., Yamin, H.G., Kellner, V., Spires-Jones, T.L., Cohen, D., and Stern, E.A. (2015). Pathological tau disrupts ongoing network activity. Neuron *85*, 959–966.

Murmu, R.P., Li, W., Holtmaat, A., and Li, J.-Y. (2013). Dendritic spine instability leads to progressive neocortical spine loss in a mouse model of Huntington's disease. J. Neurosci. *33*, 12997–13009.

Petersen, C.C.H., and Crochet, S. (2013). Synaptic computation and sensory processing in neocortical layer 2/3. Neuron *78*, 28–48.

Ramsden, M., Kotilinek, L., Forster, C., Paulson, J., McGowan, E., SantaCruz, K., Guimaraes, A., Yue, M., Lewis, J., Carlson, G., et al. (2005). Age-dependent neurofibrillary tangle formation, neuron loss, and memory impairment in a mouse model of human tauopathy (P301L). J. Neurosci. *25*, 10637–10647.

Rochefort, N.L., and Konnerth, A. (2012). Dendritic spines: from structure to in vivo function. EMBO Rep. *13*, 699–708.

Rocher, A.B., Crimins, J.L., Amatrudo, J.M., Kinson, M.S., Todd-Brown, M.A., Lewis, J., and Luebke, J.I. (2010). Structural and functional changes in tau mutant mice neurons are not linked to the presence of NFTs. Exp. Neurol. *223*, 385–393.

Sala, C., and Segal, M. (2014). Dendritic spines: the locus of structural and functional plasticity. Physiol. Rev. *94*, 141–188.

Santacruz, K., Lewis, J., Spires, T., Paulson, J., Kotilinek, L., Ingelsson, M., Guimaraes, A., DeTure, M., Ramsden, M., McGowan, E., et al. (2005). Tau suppression in a neurodegenerative mouse model improves memory function. Science *309*, 476–481.

Scheff, S.W., Price, D.A., Schmitt, F.A., and Mufson, E.J. (2006). Hippocampal synaptic loss in early Alzheimer's disease and mild cognitive impairment. Neurobiol. Aging *27*, 1372–1384.

Shepherd, G.M.G., Stepanyants, A., Bureau, I., Chklovskii, D., and Svoboda, K. (2005). Geometric and functional organization of cortical circuits. Nat. Neurosci. *8*, 782–790.

Sperling, R.A., Aisen, P.S., Beckett, L.A., Bennett, D.A., Craft, S., Fagan, A.M., Iwatsubo, T., Jack, C.R., Jr., Kaye, J., Montine, T.J., et al. (2011). Toward defining the preclinical stages of Alzheimer's disease: recommendations from the National Institute on Aging-Alzheimer's Association workgroups on diagnostic guidelines for Alzheimer's disease. Alzheimers Dement. *7*, 280–292.

Spires, T.L., Orne, J.D., SantaCruz, K., Pitstick, R., Carlson, G.A., Ashe, K.H., and Hyman, B.T. (2006). Region-specific dissociation of neuronal loss and neurofibrillary pathology in a mouse model of tauopathy. Am. J. Pathol. *168*, 1598–1607.

Spires-Jones, T., and Knafo, S. (2012). Spines, plasticity, and cognition in Alzheimer's model mice. Neural Plast. *2012*, 319836.

Trachtenberg, J.T., Chen, B.E., Knott, G.W., Feng, G., Sanes, J.R., Welker, E., and Svoboda, K. (2002). Long-term in vivo imaging of experience-dependent synaptic plasticity in adult cortex. Nature *420*, 788–794.

Cell Reports, Volume *18*

Supplemental Information

Altered Synapse Stability

in the Early Stages of Tauopathy

Johanna S. Jackson, Jonathan Witton, James D. Johnson, Zeshan Ahmed, Mark Ward, Andrew D. Randall, Michael L. Hutton, John T. Isaac, Michael J. O'Neill, and Michael C. Ashby

SUPPLEMENTARY INFORMATION – "Altered synapse stability in the early stages of tauopathy", Jackson et al.

Supplementary Information Inventory

- Supplementary Experimental Procedures – detailed description of experimental and analytical procedures
- Supplementary Figure 1 (related to Figure 1) – Histopathological data related to potential impact of viral injection and cranial window implantation.
- Supplementary Figure 2 (related to Figure 3) – Example recording of online analysis of breathing rate during functional calcium imaging, and distribution of signal:noise ratio comparing genotypes from experiments in Figure 3.
- Supplementary Figure 3 (related to Figures 1&3) – Generalized Linear Mixed Models to assess the potential impact of "between-animal" differences on structural (related to Figure 1) and functional (related to Figure 3) datasets.

Supplementary Experimental procedures

Surgery, imaging and image analysis were performed blind with respect to genotype and age.

Animals

Adult male mice of the rTg(tau_{301L})4510 (TG; n=14) line and wild-type littermate controls (WT;n=16) were used between 18 and 29 weeks of age (Ramsden et al., 2005). Mice were screened for activator and responder transgenes using a standardized PCR assay (Santacruz et al., 2005). Although this transgene contains a conditional expression cassette, mice were maintained off doxycycline-containing diet throughout, meaning mutant tau was continually expressed (Santacruz et al., 2005). All mice were given ad libitum access to food and water and maintained in a 12 hour light-dark cycle. All procedures were conducted by researchers holding a UK personal licence and conducted in accordance with the UK Animals (Scientific Procedures) Act 1986 and subject to internal ethical review.

Surgery

Cranial windows were surgically implanted over the somatosensory cortex as previously described (Holtmaat et al., 2012). In addition, adeno-associated virus expressing GFP or GCaMP6m was injected into the layer 2/3 of the cerebral cortex to enable the visualisation of cortical neurons. Briefly, mice were anaesthetized with isofluorane and administered dexamethasone (30 mg/kg) to limit brain swelling and the analgesic, buprenorphine (5 mg/kg) pre-operatively. The skull was exposed and a 5 mm diameter craniotomy drilled over the somatosensory cortex. For neurite imaging experiments AAV serotype 2 expressing GFP (10^{10} GU; Vector Biolabs), or, for neuronal activity imaging experiments, AAV (1/2 hybrid serotype) containing GCaMP6m (Chen et al., 2013) under control of the human synapsin promoter (UPenn Vector Core) was injected into layer 2/3 of the cerebral cortex (3 injection sites, 0.3µl per site, 300µm below the dura). A glass coverslip was placed over the craniotomy and sealed with glue and dental cement. A screw was placed in the skull on the contralateral side for added stability. The whole skull was subsequently covered in dental cement and a stainless steel head-post (<500 mg) was affixed to the skull contralateral to the cranial window using bone cement for positioning on the two-photon microscope. Mice were allowed to recover for at least 3 weeks before imaging began. Pilot studies showed that the injected virus diffused in the cortex and transduced cells in all cortical layers in both groups of animals.

Immunohistochemistry

Brains were coronally dissected into three segments using an adult mouse brain matrix (slot #5 and #11 AP; RBM-2000C: ASI Instruments, USA). These segments were processed using the Tissue TEK® VIP processor (GMI Inc, USA) and embedded in paraffin wax. The middle segment was used to cut 8 µm serial sections using rotary microtomes (HM 200 and HM 355; Thermo Scientific, Germany) which were mounted on glass slides. Coronal sections representing approximately Bregma -1.50 (AP) were selected for immunohistochemistry using primary antibodies specific for phospho-tau (PG-5, tau phosphorylated at s409; 1:8000, from

Peter Davies) and microglia (Iba-1: 1:6000, Wako Chemicals GmbH, Germany). Following de-paraffinisation and rehydration of the tissue, antigen retrieval was performed using the Lab Vision PT module system (Thermo Scientific, UK), where sections were heated to 100°C for 20 min in citrate buffer (TA-250-PM1X; Thermo Scientific, UK). After cooling in dH_2O, the slides were transferred to the Lab Vision Autostainer 360 (Thermo Scientific, UK) where the following incubations were performed: 10 min in H2O2 (0.03%); 30 min in normal goat serum (1:20; Vector Laboratories, USA); 60 min in primary antibodies; 30 min in biotinylated goat anti-mouse or anti-rabbit IgG (1:200, PA-920 or BA-1000; Vector Laboratories, USA); 30 min avidin-biotin complex solution (PK-7100; Vector Laboratories, USA); 5 min in 3,3′-diaminobenzidine (SK-4105; Vector Laboratories, USA). Apart from the last two steps, PBS with 0.05% Tween-20 (PBS-T) was used for diluting reagents and washes between steps. Sections were then counterstained with haematoxylin before dehydration and cover-slipping.

Neuropathological analysis

Stained sections were digitised using the Scanscope XT slide scanner (Aperio, USA) at 20× magnification. Imagescope software (version 11.1.2.760; Aperio, USA) was used to view the digitised tissue sections and delineate boundaries of the hippocampus and overlying cortex (including barrel field of the somatosensory cortex) for both the right (cranial window) and left (contralateral) side of brain. Immunoreactivity for PG-5 positive tau pathology and Iba-1 positive microglia within the regions of interest was quantified using the positive pixel algorithm (Imagescope, version 11.1.2.760; Aperio, CA, USA) and expressed as a percentage of the total area. Levels of Iba-1 and PG-5 in cortical regions were normalized to their respective levels in the hippocampus. Injection and cranial window implantation did trigger an increase in microglial activation, measured by Iba-1 immunostaining, in the ipsilateral cortex. However, this both WT and rTg4510 mice were equally prone to this elevation (**Figure S1**).

There was no difference in tau burden in cortex ipsilateral to the cranial window compared to the contralateral hemisphere (**Figure S1**).

Two-photon imaging of neurite structure

Three groups of animals were imaged; an early group imaged between 18 and 19 weeks of age, a middle group imaged between 21 and 25 weeks, and a later group imaged between 25 and 29 weeks. All time points are prior to the onset of widespread cell death in the cortex.

A two-photon microscope (Prairie Technologies) equipped with a tuneable Ti:Sapphire pulsed laser (~100 fs pulsewidth, MaiTai HP, SpectraPhysics) and PrairieView acquisition software was used for all imaging experiments. Mice were anaesthetized with 3-5% isoflurane and secured to the microscope with the metal bar attached to the skull to a custom-built fixed support. Body temperature of 35-37°C was maintained by a heating blanket and rectal probe. A 10x objective (NA=0.3, Olympus) was used to identify characteristic blood vessels to reliably locate regions-of-interest (ROIs) at each imaging time point. A 40x water immersion objective (LUMPlanFI/IR, NA=0.8, Olympus) was used to acquire several ROI stacks (75 μm x 75 μm, 512 x 512 pixels, step size = 0.5 μm) per animal. Excitation power at 910 nm was typically 35mW at the sample. GFP emission was collected through a 525/25nm filter. At the first imaging session, 5-10 ROIs of axons and/or dendrites were acquired ensuring that the ROIs were at least 100 μm apart. Animals underwent subsequent imaging sessions, each lasting approximately one hour, up to a maximum of 20 sessions. The signal-to-noise ratio was measured in WT and rTg4510 images and no significant difference was found.

Widefield epifluorescence imaging of neuronal activity

22 week-old mice were head-fixed under sedation (2 mg/kg cholorprothixene) and light, constant anaesthesia (0.4-0.8 % isoflurane). Body temperature of 35-37°C was maintained by a heating blanket and rectal probe. Barrel locations were mapped in S1 via widefield imaging

of neuronal GCaMP6m fluorescence (Minderer et al., 2012). Individual whiskers were loaded into a 19 gauge cannula to approximately 5 mm from the snout, and deflected caudo-rostrally (5° deflections, 10 Hz, 2 s) using a piezo bending actuator (Piezo Systems, Woburn, MA). Neuronally-expressed GCaMP6m was excited by illuminating the cortical surface with 480 nm light and epifluorescence images were captured through a 500-550nm bandpass emission filter at 10 Hz using a 4x objective (Plan N, Olympus, NA 0.1) and 12-bit CCD camera (QICAM Fast 1394, QImaging). Stimulation-evoked responses were calculated per pixel per trial (10 trials, 20 s inter-trial interval) as the fractional change in mean GCaMP6m fluorescence 0-2 s post whisker-stimulation onset relative a 0-2 s pre-stimulation baseline. Response fields were determined as the largest contiguous area in which the in which the response amplitude exceeded 50% of the maximum response. Regions within the response field were subsequently targeted for higher magnification two-photon imaging.

Two-photon imaging of neuronal activity

Images were acquired at 40x objective magnification (LUMPlanFI/IR, 0.8 NA, Olympus) using a two-photon microscope (Scientifica), equipped with Ti:sapphire pulsed laser (~100 fs pulsewidth, Mai Tai-HP, Newport Spectra-Physics) and galvanometric scan mirrors. GCaMP6m was excited at 910 nm and fluorescence collected using a 525/25 nm emission filter. Excitation power was <35mW at the sample Images were acquired using bidirectional frame scanning at 8 Hz, 256 x 128 pixel resolution, using ScanImage 3.8 software (Pologruto et al., 2003). Superficial cortical blood vessels were used as fiducial markers to guide imaging to regions as close as possible to the principal whisker response field that contained a high density of GCaMP6m expressing layer 2/3 neurons (100-300 µm below pial surface) and did not overlap with major blood vessels. A high-resolution (0.15 µm per pixel) reference image was acquired of each imaging field to assist post-hoc definition of neuronal regions of interest (ROIs). Spontaneous GCaMP6m signals were recorded continuously for 520 s. For whisker

stimulation experiments, images were acquired continuously while the principal whisker was deflected caudo-rostrally (5° deflections, 10 Hz, 1 s) at a 20 s inter-stimulus interval across 25 trials using a piezo actuator. To ensure that a consistent depth of anaesthesia was maintained throughout and between experiments, breathing rate was continuously monitored using a piezoelectric wafer beneath the thorax, and anaesthesia adjusted to maintain a rate >100 breaths per min (**Figure S2B-D**) (Golshani and Portera-Cailliau, 2008). In separate experiments, we determined that small variations within the range of breathing rates used here (100-140 breaths per min) did not correlate with the rate or amplitude of spontaneous neuronal activity. Deeper anaesthesia, and associated slower breathing rate, dramatically reduced apparent excitability (data not shown).

Spine and bouton structure analysis

In vivo two-photon images were converted into stacks with ImageJ and the StackReg plugin run to align the stacks in case of any movement. The stacks were deconvolved with Huygens Deconvolution software using a quick maximum likelihood estimation with an experimentally defined point-spread-function. Deconvolved stacks were then imported into Neurolucida and the density of dendritic spines or axonal boutons determined. To confirm that a fluorescent protrusion was attached to the dendritic/axonal shaft, the brightness and contrast of the image was adjusted to see a visible spine/bouton neck. Analysts distinguished between dendritic spines and axonal TBs by their attachment to a thicker straighter dendritic shaft compared to the thinner, more tortuous, axonal process, as described in the existing literature (De Paola et al., 2006; Majewska et al., 2006). No changes were observed in the proportion of dendritic or axonal filopodia at any time point (data not shown). For analysis of turnover, the image stacks were imported into ImageJ and spines or boutons marked using the Cell Counter plugin for each imaging session. These parameters were then further analysed in Microsoft Excel and statistics carried out in SigmaPlot. Turnover ratio (TOR) was determined by TOR (t_1,t_2) =

$(N_{gained} + N_{lost})/N(t_1) + N(t_2)$ where $N(t_1)$ and $N(t_2)$ are the total number of synaptic structures at the first and second time point respectively. Survival fraction was calculated as $SF(t) = N(t)/N_0$ where N_0 is the number of structures at t=0 and N(t) if the number of structures of present at the first imaging session surviving after time t. Persistent and transient spines/boutons were defined as ones there for at least two imaging sessions (>8days) or only one imaging session (<8 days) respectively. For animals where axons and dendrites were imaged, the TOR, gains and losses and persistent fraction was compared, using a Student's *t* test, between axons and dendrites in both WT and rTg4510 groups. Two-way repeated measures ANOVA tests were used with the Holm-Sidak method of pairwise comparison in all analyses to determine significance when $p < 0.05$ and where *n* was the number of axons or dendrites. Only those axons/dendrites which were present for two imaging sessions at the early time point and >3 imaging sessions in the mid and late time points were used. The size of TB or spine heads was measured as the diameter of a manually-drawn circle that bounded the widest part of the structure (in deconvolved image stacks). Individual measurements were grouped based on age and genotype and statistically tested using a Generalized Linear Model (SPSS, IBM).

Neuronal activity analysis

Analyses were performed using Matlab (Mathworks) and ImageJ software (National Institutes of Health). Mechanical drift was corrected by registration to the average of the first 5 seconds (40 frames) using the TurboReg plugin (Thevenaz et al., 1998) in ImageJ. Summed and standard deviation projections of time-lapse images were used to manually draw ROIs around visually identified neuronal cell bodies.

For analysis of spontaneous GCaMP6m signals, neuronal fluorescence time series, *F(t)*, were constructed by averaging the ROI pixels in each frame. The background time series was

determined from a reference blood vessel and was subtracted from *F(t)*. Relative time series were generated following the procedure of Jia et al. (Jia et al., 2011). Briefly, a time-varying baseline, $F_0(t)$, was determined by first smoothing *F(t)* with a ±1 s moving average filter to generate *F'(t),* and then for each time point, *t*, F_0 was calculated as the minimum of *F'(t)* in the preceding 5 second epoch, such that:

$$F_0(t) = \{\min(\mathrm{F'(x)}) \mid (t-5) < x < t\}$$

where $F'(x) = \frac{1}{2}\int_{x-1}^{x+1} F(\tau)\mathrm{d}(\tau)$

The relative fluorescence time series was then calculated as:

$$R(t) = \frac{F(t) - F_0(t)}{F_0(t)}$$

Finally, the relative time series was smoothed with an exponentially weighted moving average to generate *ΔF/F*:

$$\Delta F/F(t) = \frac{\int_0^t R(t-\tau)\cdot w(\tau)\mathrm{d}\tau}{\int_0^t w(\tau)\mathrm{d}\tau}$$

where $w(\tau) = exp\left\{-\frac{|\tau|}{0.5}\right\}$

This routine is robust against slow drifts in baseline fluorescence (Jia et al., 2011).

Spontaneous fluorescence transients were isolated using a high-throughput template-based event detection algorithm, following the methodology of Clements and Bekkers (Clements and Bekkers, 1997). A template, *T*, was constructed by averaging and smoothing (0.5 s moving average) the waveforms of 310 manually approved events (aligned to event onset) from WT neurons (n=3 mice, 5 sessions). The minima and maxima of *T* were normalised to 0 and 1

respectively. For each ΔF/F time series, *T* was advanced point-by-point along the time series and the fit, *Q*, calculated as:

$$Q = T * S + P$$

where *T* is the template, *S* is the template scaling factor, and *P* is the offset:

$$S = \frac{\sum(T * D) - \sum T * \sum D/N}{\sum T^2 - \sum T * \sum T/N}$$

$$P = \frac{\sum D - S * \sum T}{N}$$

where N is the number of samples in the template, Σ is the sum from 1 to N, and *D* is the period of the ΔF/F time series to fit, such that:

$$D = \{\Delta\,\mathrm{F/F(x)} \mid t \le x \le (t + N - 1)\}$$

The detection criterion, *C*, at each time point, *t*, was then calculated from *S* and the standard error of the fit, *E*:

$$E = \sqrt{\frac{\sum(D - Q)^2}{N - 1}}$$

such that

$$C(t) = \frac{S}{E}$$

This algorithm directly relates the detection criterion to the signal-to-noise ratio of the fit, thus accounting for variable levels of noise within and between neuronal time series (Clements and Bekkers, 1997). Events were detected at times, *t*, when *C(t)* exceeded a threshold of 2. Event rise times were calculated using the 20-80 % portion of the initial rising phase of the event.

For analysis of whisker stimulation-evoked responses, neuronal fluorescence time series were constructed and background corrected. The time series were binned into 10 s whisker

stimulation trial epochs (WS) and interleaved 10 s non-whisker stimulation trial epochs (NS), and relative fluorescence time series computed for individual WS and NS with baseline fluorescence, F_0, dynamically calculated as the mean of the 2 s pre stimulus period, such that:

$$\Delta F/F_0(t) = \frac{F(t)-F_0}{F_0}$$

Neuronal WS responses were defined as the integral of the ΔF/F time series 0-2 s post whisker stimulation onset. Neurons were classified as responsive to the whisker stimulus if, (1) the distribution of WS responses was significantly greater than the distribution of NS responses (determined using a 2-sample KS-test), and (2) the peak amplitude of the trial averaged response (0-2 post stimulation) exceeded 4 times the standard deviation of the trial averaged baseline (0-2 pre stimulation). This stringent detection criterion generated zero false positives, based on post-hoc visual inspection of the individual WS and NS traces.

Comparison of the signal-to-noise ratio of image regions from individual cells (calculated following the protocol of Kerlin et al. (2010)) showed that there was no difference in the population distribution between WT and rTg4510 animals (**Figure S2A**). This suggests that the imaging preparation and GCaMP6m expression are comparable across genotype.

Statistical testing was performed using SPSS (IBM) and Matlab. Datasets were tested for normality and parametric or nonparametric tests used as appropriate with an α level of 0.05. Data are presented as mean ± SEM unless otherwise stated. n numbers for statistical testing are noted as “n=…” throughout.

Statistical analysis of effects of individual animals

In all statistical analyses (except Figures 2C&F, where spine and TB results are compared within animals), the cell (or cellular events in Figure 3D) was chosen as our primary dependent variable (or “n” number). This level of analysis was used (in particular, avoiding averaging

within each animal) because we were particularly interested in the variability between individual cells or neurites. This is because neurodegeneration is a slowly progressing process that affects different neurons at different times. It is changes in the characteristics of this population distribution that are likely to be a primary indicator of potential disease effects. However, cells within an individual animal are not independent and may have a tendency to co-vary, which could generate bias within our datasets. To minimize potential difficulties due to unevenly weighted contributions of individual animals, balanced numbers of cells/neurites from each animal were included in each sample. Further, to formally test the effect of the animal on our datasets, Generalized Linear Mixed Models (GLMM, SPSS) were generated that include "Genotype" as a Fixed factor and "Animal" as a Random factor, allowing by-neuron random intercept, and by-animal random slope and by-genotype random slope. When repeated measures were made (eg, in longitudinal spine density measures), "Age" was also included as a Fixed factor, using a first-order autoregressive (AR1) covariance structure because we anticipate higher covariance between ages that are closer in time. Axon/spine density (**Figure 1**) and cellular activity (**Figure 3**) values were modelled in the GLMM with a gamma distribution and log link function, since population distributions tended to be skewed to higher values.

Results of GLMMs of our structural (spine and TB density) and functional (spontaneous activity) datasets are presented in **Figure S3**. The effects of genotype in the GLMM are established by the "genotype coefficient" (**Figure S3A**), which indicates the average effect of being Tg4510-positive on spine or TB density. For example, Tg4510 dendrites in the late age group will, on average, have 0.594 spines/μm^{-1} less than WT counterparts (**Figure S3A**). The variance in the GLMM that is due to animal-related effects is measured by the "variance due to animal" metric. Significance values for these predicted effects are shown in the final two columns of **Figure S3A** (green highlights $p<0.05$). In line with analyses presented in Figure 1,

significant decreases due to the transgene are found in spine density in the mid and late age groups (**Figure S3B**) and in TB density in the late group (**Figure S3C**). In none of the datasets was there a significant effect of within-animal co-variance (**Figure S3A-C**). Similar GLMMs were used to analyse the spontaneous neuronal calcium elevations recorded in vivo (shown in **Figure 3A-D**). In these analyses, being Tg4510-positive is associated with a significant reduction in the frequency, ampltiude and rise time of spontaneous events (**Figure S3D-G**). Again, in these data, the effect of individual animal identity had no significant influence on the GLMM predictions (**Figure S3D-G**). This quantitatively defines the contribution of animal-to-animal variability as having a limited impact on our findings. We were not able to obtain well-fitted GLMMs for the binomial data for response to whisker stimulation (**Figure 3E-F**), possibly because the fraction of responding cells is relatively small due to the sparse coding of such sensory stimuli in this neuronal population.

Supplementary References

Clements, J.D., and Bekkers, J.M. (1997). Detection of spontaneous synaptic events with an optimally scaled template. Biophys. J. *73*, 220–229.

De Paola, V., Holtmaat, A., Knott, G., Song, S., Wilbrecht, L., Caroni, P., and Svoboda, K. (2006). Cell type-specific structural plasticity of axonal branches and boutons in the adult neocortex. Neuron *49*, 861–875.

Golshani, P., and Portera-Cailliau, C. (2008). In Vivo 2-Photon Calcium Imaging in Layer 2/3 of Mice. J. Vis. Exp. JoVE.

Holtmaat, A., de Paola, V., Wilbrecht, L., Trachtenberg, J.T., Svoboda, K., and Portera-Cailliau, C. (2012). Imaging neocortical neurons through a chronic cranial window. Cold Spring Harb. Protoc. *2012*, 694–701.

Jia, H., Rochefort, N.L., Chen, X., and Konnerth, A. (2011). In vivo two-photon imaging of sensory-evoked dendritic calcium signals in cortical neurons. Nat. Protoc. *6*, 28–35.

Kerlin, A.M., Andermann, M.L., Berezovskii, V.K., and Reid, R.C. (2010). Broadly Tuned Response Properties of Diverse Inhibitory Neuron Subtypes in Mouse Visual Cortex. Neuron *67*, 858–871.

Majewska, A.K., Newton, J.R., and Sur, M. (2006). Remodeling of Synaptic Structure in Sensory Cortical Areas In Vivo. J. Neurosci. *26*, 3021–3029.

Minderer, M., Liu, W., Sumanovski, L.T., Kügler, S., Helmchen, F., and Margolis, D.J. (2012). Chronic imaging of cortical sensory map dynamics using a genetically encoded calcium indicator. J. Physiol. *590*, 99–107.

Pologruto, T.A., Sabatini, B.L., and Svoboda, K. (2003). ScanImage: flexible software for operating laser scanning microscopes. Biomed. Eng. Online *2*, 13.

Ramsden, M., Kotilinek, L., Forster, C., Paulson, J., McGowan, E., SantaCruz, K., Guimaraes, A., Yue, M., Lewis, J., Carlson, G., et al. (2005). Age-dependent neurofibrillary tangle formation, neuron loss, and memory impairment in a mouse model of human tauopathy (P301L). J. Neurosci. *25*, 10637–10647.

Santacruz, K., Lewis, J., Spires, T., Paulson, J., Kotilinek, L., Ingelsson, M., Guimaraes, A., DeTure, M., Ramsden, M., McGowan, E., et al. (2005). Tau suppression in a neurodegenerative mouse model improves memory function. Science *309*, 476–481.

Thevenaz, P., Ruttimann, U.E., and Unser, M. (1998). A pyramid approach to subpixel registration based on intensity. IEEE Trans. Image Process. *7*, 27–41.

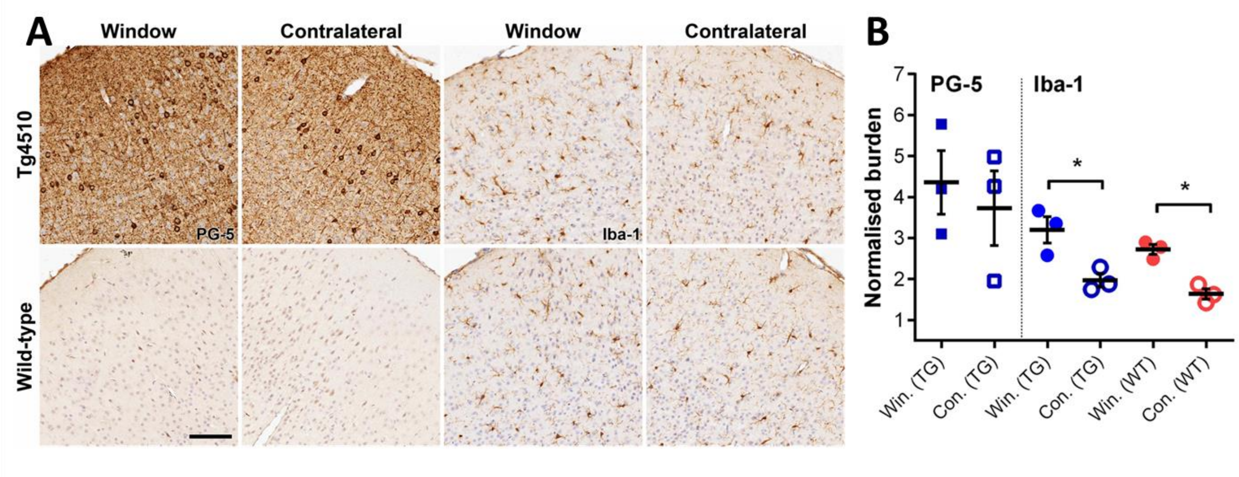

Figure S1 (related to Figure 1): Virus injection and/or cranial window implantation does not cause abnormal pathology in rTg4510 animals at six weeks post-surgery.

(A) Representative images of phosphorylated tau expression (PG5) in the somatosensory cortex beneath the window and in the contralateral hemisphere in WT and rTg4510 animals (left four panels). Representative images of microglial (Iba-1) response to the virus injection and cranial window implantation compared to the contralateral hemisphere in both groups of animals (right four panels). (B) No significant difference was seen in phosphorylated tau levels between the ipsilateral and contralateral hemispheres. However, the somatosensory cortex beneath the window did have a significant increase in microgliosis compared to the contralateral hemisphere in both groups of animals ($F_{(3,8)} = 12.38$; p=0.002; WT: n=3, rTg4510: n=3), B(PG-5): One-way ANOVA, B(Iba-1): Student's unpaired t test. *p<0.05. Error bars = mean ± SEM. Scale bar = 100µm.

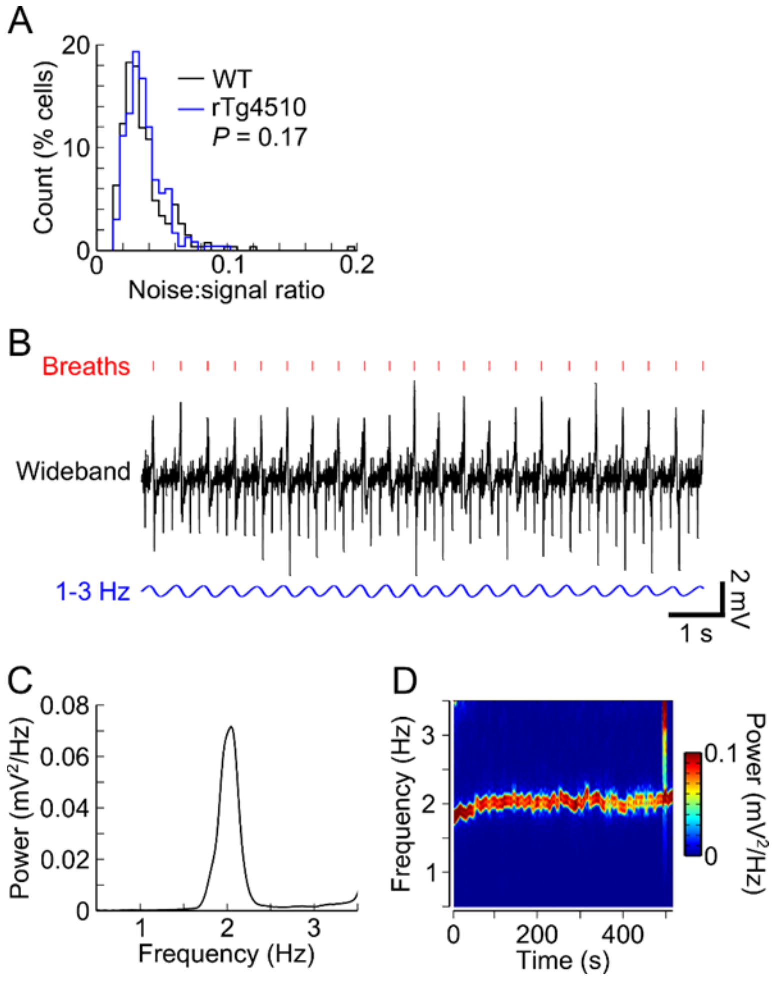

Figure S2 (related to Figure 3): Imaging conditions were consistent throughout functional calcium imaging experiments and between genotypes.

(A) Histograms illustrating consistent fluorescence noise-to-signal ratios between WT (n=268, 3 mice) and rTg4510 (n=233, 3 mice) GCaMP6m-expressing neurons imaged during two-photon imaging experiments (p=0.17; rank-sum test). Ratios were determined by estimating the contribution of photon shot noise to the mean neuronal fluorescence signal, as described by Kerlin et al. (2010). **(B)** Representative 10 s wideband (black) and 1-3 Hz bandpass filtered (blue) traces of breathing movements recorded in a WT mouse during a two-photon imaging experiment using a piezoelectric transducer beneath the thorax. Large amplitude transients (red ticks) correspond to individual breaths. Smaller amplitude, higher frequency transients correspond to heart beats. **(C)** Power spectral density of the wideband trace in (a) between 0.5-

3.5 Hz, illustrating a 2 Hz breathing rate. **(D)** Spectrogram of the full breathing movement trace in (a) across a 520 s time-lapse imaging experiment. A stable ~2 Hz breathing rate was maintained throughout the experiment.

A

dataset		Tg4510 coefficient	variance due to animal	genotype p value	animal p value
spines	Early	-0.167	0.056	0.18	0.16
spines	Mid	-0.374	0.056	0.001	0.054
spines	Late	-0.594	0.117	0.000045	0.082
TBs	Early	0.002	0.057	0.99	0.099
TBs	Mid	-0.243	0.237	0.417	0.142
TBs	Late	-0.819	0.264	0.02	0.137

B

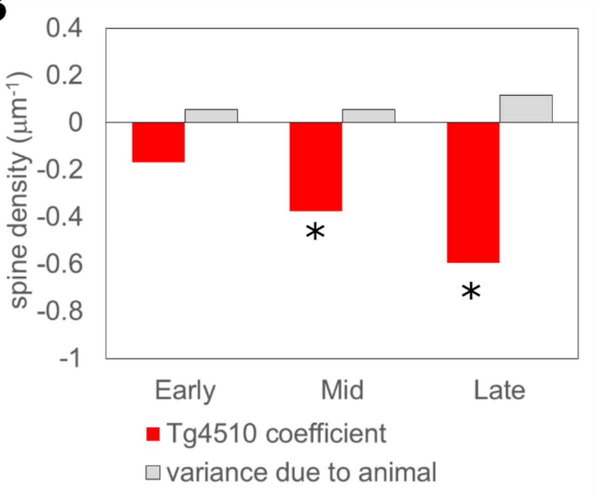

C

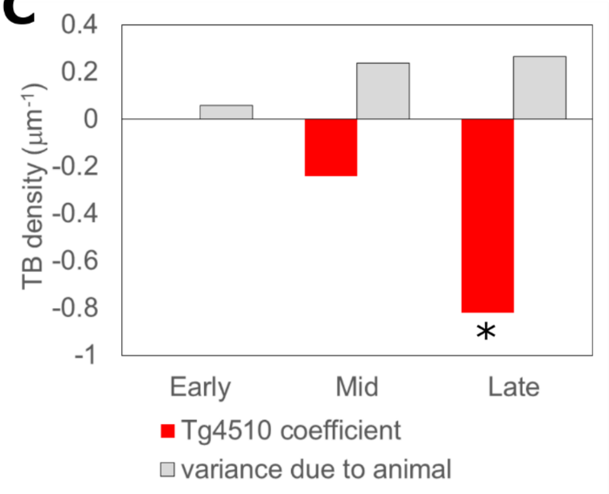

D

analysis	Tg4510 coefficient	variance due to animal	genotype p value	animal p value
event frequency (Hz)	-1.957	0.372	0.0003	0.18
event amplitude ($\Delta F/F_0$)	-0.182	0.072	0.016	0.403
event rise time (ms)	-0.413	0.129	0.018	0.888

E

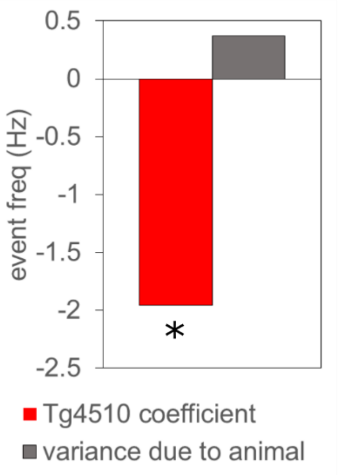

F

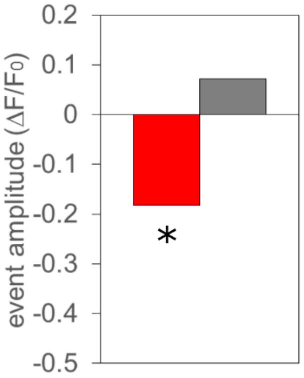

G

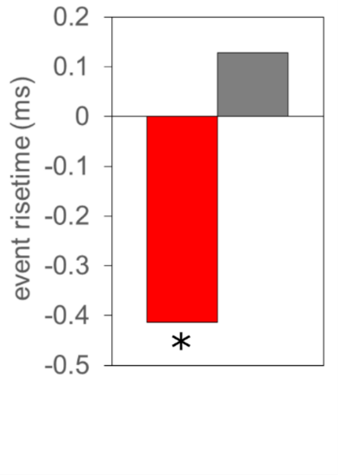

Figure S3 (related to Figures 1&3): Results of GLMM showing that animal-to-animal variance has small and non-significant effects on the primary measures

(A) Table showing the results of GLMM of synapse density for dendritic spines and axonal TBs^{--} in the early, mid and late age groups. Coefficients of genotype (effect of being Tg4510-positive relative to WT) and average variance associated with animal are shown, alongside p values. Dataset as in Figure 1. (B) Histograms showing the effect of being Tg4510-positive on spine density, alongside variance associated with the animal. (C) Histogram showing the effect of being Tg4510-positive on TB density, alongside variance associated with the animal. (D) Table showing the results of GLMMs of properties (frequency, amplitude, rise time) of spontaneous calcium elevations (dataset as in Figure 3A-D). Coefficients of genotype and average variance associated with animal are shown, alongside p values. (E-G) Histograms showing the showing the effect of being Tg4510-positive on the frequency (E), amplitude (F) and rise time (G) of spontaneous calcium events, alongside variance associated with the animal in each case. (* indicates $p<0.05$, highlighted in green in tables). (E)
